# Self-supporting graphitized palm-branch anodes for enhanced electricity generation from sewage wastewater in membrane-less microbial fuel cells

**DOI:** 10.1098/rsos.261016

**Published:** 2026-10-21

**Authors:** Nasser A. M. Barakat, Mahmoud M. Bin, Ibrahim Ashour

**Affiliations:** ^1^ Chemical Engineering Department, College of Engineering, King Faisal University, P.O. Box 380, Al-Ahsa 31982, Saudi Arabia; ^2^ Chemical Engineering Department, Minia University, Minya, Egypt

**Keywords:** microbial fuel cell, graphitized palm tree branches, self-supporting anode, sewage wastewater, bioelectricity generation

## Abstract

A simple strategy was developed to fabricate self-supporting bioanodes from graphitized palm-tree-branch powder for application in membrane-less microbial fuel cells (MFCs). Palm tree branches were graphitized at 900°C, 1000°C and 1100°C, ground into fine powder, blended with bitumen and starch, compression-moulded and thermally treated to produce robust anodes. The resulting electrodes were evaluated in an air-cathode, single-chamber MFC using raw sewage wastewater without external microbial inoculation or nutrient addition. Structural and electrochemical analyses showed that the prepared anodes possessed rough porous surfaces, stable carbon-dominated electrochemical behaviour and improved conductivity compared with an activated-carbon-based anode. Among the investigated samples, the anode prepared from palm branches graphitized at 1000°C exhibited the best overall performance. In sewage containing 5g l^−1^ sodium acetate, this anode delivered a maximum power density of about 1300 mW m^−2^, which was nearly 25 times higher than that of the activated-carbon-based anode (approx. 52 mW m^−2^). Even under electrolyte-free conditions, the same anode generated a maximum power density of about 400 mW m^−2^, indicating that intrinsically retained inorganic species, particularly KCl, may contribute to sustaining MFC operation without external electrolyte addition. In contrast, NaCl showed a much weaker promoting effect than sodium acetate. Electrochemical impedance spectroscopy confirmed relatively low ohmic and charge-transfer resistances under the optimum condition.

## 1. Introduction

Recovering useful energy during wastewater treatment has emerged as one of the most compelling strategies for addressing the intertwined challenges of environmental pollution and growing energy demand. In this context, microbial fuel cells (MFCs) have attracted increasing attention as bioelectrochemical systems capable of directly converting the chemical energy stored in biodegradable organic matter into electricity while simultaneously contributing to wastewater remediation [1]. Unlike conventional treatment technologies that rely heavily on external energy input and generate large amounts of excess sludge, MFCs offer the possibility of a more sustainable treatment route in which waste streams are viewed as energy-bearing resources rather than disposal burdens [2]. This dual functionality has made MFC technology particularly appealing for decentralized and low-energy treatment applications, especially where simple system design and resource recovery are of primary importance [3]. Nevertheless, translating this promise into practical implementation requires continued advances in both cell architecture and electrode materials [4].

Despite this promising concept, the practical deployment of MFC technology remains constrained by several persistent challenges, including low power output, high internal resistance, slow extracellular electron transfer and the relatively high cost of electrochemical components [5]. These limitations become even more pronounced under real wastewater conditions, where fluctuations in organic composition, ionic strength and microbial activity can markedly influence cell performance and stability [6]. Among the different factors governing MFC efficiency, electrode design has been recognized as one of the most decisive, because the electrodes control both the electrochemical reactions and the interaction between the microbial community and the conductive surface [7]. In particular, the anode plays a central role in determining the effectiveness of bacterial adhesion, biofilm development, substrate oxidation and electron harvesting [8,9]. Therefore, improving anode materials and fabrication strategies is not merely a matter of material substitution but a key step towards narrowing the gap between laboratory-scale demonstrations and practical wastewater-based MFC applications.

An ideal anode should therefore combine high conductivity with a sufficiently large and accessible surface area, structural robustness, biocompatibility and low cost. Carbon-based materials have been widely preferred for this purpose because of their favourable conductivity, chemical stability and resistance to corrosion; however, many commercially available carbon electrodes remain relatively expensive or require additional modification to achieve satisfactory performance [10,11].

In this regard, biomass-derived carbon materials have emerged as highly attractive candidates for MFC anodes owing to their low cost, renewability, environmental compatibility and rich structural diversity [12,13]. A wide range of agricultural and forestry residues can be converted into carbonaceous materials with tunable pore structure, surface chemistry and morphology, making them suitable for bioelectrochemical applications [14–17]. More importantly, when such biomass precursors undergo graphitization, the resulting carbon framework can exhibit substantially enhanced electrical conductivity compared with conventional biochar, thereby improving charge transport within the anode body. This combination of sustainability and functional performance makes graphitized biomass particularly appealing for the development of next-generation MFC electrodes [18,19].

Among the various biomass resources, palm tree residues represent a particularly attractive yet still insufficiently exploited precursor for the production of conductive carbon materials. Converting palm tree branches into graphitized carbon is especially appealing because it combines waste valorization with the generation of a low-cost electrode material that can be integrated into sustainable energy-recovery systems [20,21]. Although biomass-derived carbons have been increasingly investigated for MFC applications, the use of graphitized palm tree branch powder as a basis for constructing a moulded, self-supporting anode remains largely unexplored.

A critical limitation in this field is that many biomass-derived carbon materials are obtained in powder form, which, despite their attractive surface and electrical properties, cannot be directly employed as practical anodes without an effective shaping and consolidation strategy. In many reported studies, such carbons are deposited onto commercial substrates, mixed with polymeric binders or used as modifiers rather than being transformed into independent electrode bodies [22–24]. While these approaches can enhance performance, they do not fully address the need for simple, low-cost and scalable manufacturing routes capable of converting graphitized biomass powders into mechanically robust, self-supporting anodes. This challenge highlights an important yet underexplored research direction in MFC technology: the development of generalizable powder-to-anode fabrication strategies that combine structural integrity, electrical continuity and processing simplicity. From this perspective, the integration of graphitized biomass powder with suitable carbonizable binders, followed by compression moulding and thermal consolidation, offers a promising route for producing shaped anodes that may be extended beyond a single biomass source to a broader range of graphitized agricultural residues.

Many biomass-derived carbon materials are obtained in powder form and, despite their attractive surface and electrical properties, require shaping and consolidation before they can be used as practical self-supporting anodes [25,26]. Palm tree branches are particularly notable in this regard, as they contain a considerable amount of potassium-based compounds that may persist, in part, after graphitization [27]. This feature adds an additional dimension to palm-derived carbon, because the retained mineral content may contribute not only to the carbonization behaviour of the precursor but also to the electrochemical environment of the final electrode.

Although many previous investigations have demonstrated encouraging performance using synthetic electrolytes, defined substrates or externally enriched microbial cultures, such conditions do not fully represent the complexity of real wastewater treatment environments. For practical implementation, MFCs must be capable of operating efficiently in actual sewage streams, where the organic composition, ionic content and indigenous microbial population are inherently variable. In this respect, the use of raw sewage wastewater without supplementation by external microorganisms or nutrient media provides a more realistic assessment of electrode functionality and system feasibility. Moreover, employing a single-chamber, membrane-less, air-cathode configuration further enhances the practical value of the system by reducing structural complexity, lowering material cost and simplifying operation, all of which are important considerations for future scale-up and field deployment.

The present study compares the performance of the developed anode in sewage wastewater in the presence of sodium acetate and sodium chloride and without external electrolyte addition. Evaluating electrolyte-free operation provides insight into whether the retained inorganic species within the graphitized palm-derived anode can partially compensate for the absence of deliberately added salts, thereby supporting a simpler and more economical MFC configuration. In addition, the effects of graphitization temperature and operating medium were systematically investigated. Through this approach, the study aims not only to introduce a practical route for converting graphitized biomass powders into self-supporting MFC anodes but also to explore the possible contribution of intrinsically retained mineral species towards reducing dependence on externally added electrolytes.

## 2. Material and methods

### 2.1. Materials

Activated carbon was used as a reference anode material and also as the catalytic component in cathode fabrication. Poly(vinylidene fluoride) (PVDF, average *M_w_* ≈ 534 000) was employed as the polymeric binder for preparing the cathode, while multi-walled carbon nanotubes (CNTs) were incorporated to enhance its electrical conductivity. For anode fabrication, bitumen and starch were used as binder components to consolidate the graphitized biomass powder into a self-supporting electrode body. *N,N*-dimethylformamide (DMF) was used as the solvent for dissolving PVDF during cathode slurry preparation. Sodium acetate (NaAc, analytical grade) and sodium chloride (NaCl, analytical grade) were used as medium additives for performance comparison during MFC operation. Silver nitrate (AgNO_3_, analytical grade) was used for the qualitative detection of chloride ion release from the graphitized anode materials. Distilled and deionized (DI) water was used throughout the experiments for washing procedures, preparation of solutions and soaking tests. Raw sewage wastewater was collected from a local municipal source and directly employed as the anodic medium without the addition of external microbial inoculum. Unless otherwise stated, all chemicals used in this work were of analytical grade and were used as received without further purification.

### 2.2. Anode preparation

Palm tree branches were first converted into graphitized carbon at different treatment temperatures of 900°C, 1000°C and 1100°C. The thermal treatment was carried out at a heating rate of 2°C min^−1^ with a dwelling time of 5 h at the target temperature. After cooling to room temperature, the resulting graphitized product was collected and ground thoroughly. To fabricate the anodes, the graphitized palm tree branch powder was mixed with bitumen and starch as dual binder components. Because the graphitized powders obtained at different temperatures exhibited different compactability and binding behaviour, the relative contents of graphitized palm powder, bitumen and starch were optimized individually for each graphitization temperature to achieve the best possible mechanical integrity of the moulded anode. The detailed formulations used for each sample are listed in table S1 in the supplementary material.

The prepared powder mixtures were homogenized carefully and then transferred into a specially designed stainless-steel mould for shaping under compression. A photographic image of the mould is presented in figure S1A,B in the supporting material. The mould consisted of a cylindrical cavity used to receive the powder mixture, a stainless-steel pressing rod to apply compaction force and a bottom support cover to maintain the desired shape during pressing. Following compaction, the moulded anodes were subjected to a secondary thermal treatment at 500°C for 5 h. This post-moulding heat treatment was applied to stabilize the pressed body, improve interparticle adhesion and convert the binder phase into a more carbonaceous and mechanically integrated framework. A photographic image of the final prepared anode is shown in figure S1C in the supplementary material. For comparison purposes, an anode based on activated carbon was also prepared using the same general moulding concept.

### 2.3. Cathode preparation

The air cathode was fabricated by a slurry-coating method. Initially, 1 g of PVDF was dissolved in 8 ml of DMF under continuous magnetic stirring until a homogeneous binder solution was obtained. Thereafter, 0.5 g of AC was added to the PVDF solution and mixed thoroughly. To improve the electrical conductivity of the cathodic layer, 0.035 g of CNTs were incorporated into the mixture. Stirring was continued until a uniform viscous ink was formed. The prepared cathodic ink was then manually spread onto one side of a carbon cloth sheet (CC, ElectroChem, USA) to obtain an even catalytic coating layer. After coating, the electrodes were dried at 60°C for 2 h to remove the residual solvent and promote consolidation of the binder matrix. The dried cathodes were subsequently subjected to thermal treatment at 350°C to further stabilize the catalytic layer and enhance adhesion between the coated materials and the carbon cloth support [28].

### 2.4. Microbial fuel cell configuration and operation

An air-cathode, membrane-less, single-chamber MFC was employed throughout this study. The reactor body was fabricated from transparent acrylic plates and tightly fastened using stainless-steel bolts to ensure a leak-free assembly during operation. The effective working volume of the anodic chamber was approximately 50 ml. Photographic images of the reactor components and the assembled cell are presented in figure S2A–C in the supplementary material. Prior to use, the collected sewage wastewater was filtered to remove large suspended solids and debris. The filtered wastewater was then directly used as the anolyte without the addition of external microbial inoculum. To evaluate the effects of medium supplementation on MFC performance, sodium acetate (NaAc) and sodium chloride (NaCl) were added at different concentrations according to the requirements of each experiment.

The prepared moulded anode was placed inside the anodic chamber and electrically connected through a graphite rod serving as the current collector. The fabricated air cathode was fixed on one side of the reactor, with the catalytic layer facing the liquid phase and the opposite side exposed directly to air to enable the oxygen reduction reaction. After the reactor was filled with the prepared wastewater, the anolyte was purged with nitrogen gas for 5 min to remove dissolved oxygen and establish anaerobic conditions in the anodic compartment. The assembled MFCs were then operated under ambient laboratory conditions for electrochemical and power-generation measurements.

### 2.5. Material and electrochemical characterizations

The crystalline structure and phase composition of the prepared biomass-derived carbon materials were analysed by X-ray diffraction (XRD) using an X-ray diffractometer equipped with Cu Kα radiation (*λ* = 1.5406 Å), operated at 40 kV and 40 mA. Diffraction patterns were recorded over a 2*θ* range of 10–80° at a scanning rate of 2° min⁻¹. The surface morphology and microstructural characteristics of the as-prepared and used anodes were examined using scanning electron microscopy (SEM). Prior to observation, the samples were mounted on aluminium stubs using conductive carbon tape and, when required, sputter-coated with a thin gold layer to improve surface conductivity.

Electrochemical measurements were carried out using a VersaSTAT 4 potentiostat/galvanostat (AMETEK Scientific Instruments, USA). Cyclic voltammetry (CV) was performed directly on the assembled MFC in a two-electrode configuration, where the graphitized palm-tree-branch anode acted as the working electrode and the air cathode served as the counter electrode. The measurements were conducted at a scan rate of 50 mV s^−1^ for successive cycles in order to evaluate the electrochemical activity and stability of the developed anode in the sewage-based medium.

Linear sweep voltammetry (LSV) was conducted to obtain polarization curves and assess the overall electrochemical performance of the MFC. In this case, a two-electrode configuration was also adopted, with the cathode serving as the working electrode, while the anode simultaneously functioned as both the counter and reference electrode. From the obtained data, key performance parameters, including open-circuit potential, maximum current density and maximum power density, were determined.

Electrochemical impedance spectroscopy (EIS) measurements were further carried out using the same instrument to investigate the resistive and interfacial characteristics of the system. Impedance spectra were recorded over a frequency range from 100 kHz to 0.01 Hz using an AC perturbation amplitude of 5–10 mV at the open-circuit potential. EIS measurements were conducted for both the whole MFC and the isolated anode by reversing the electrode connections when needed. The resulting Nyquist plots were analysed using suitable equivalent circuit models to estimate the ohmic resistance (*R_s_*), charge-transfer resistance (*R*_ct_) and diffusion-related contributions. All electrochemical experiments were carried out at ambient laboratory temperature (25 ± 2°C), and the current and power densities were normalized to the projected surface area of the cathode. Moreover, the measurements were repeated three times under identical experimental conditions to verify reproducibility. Owing to the large number of closely spaced data points in these electrochemical profiles, representative curves from reproducible measurements are presented without point-by-point error bars to maintain graphical clarity.

## 3. Results and discussion

### 3.1. Anode characterization

#### 3.1.1. Chemical composition

The crystalline structure of the graphitized palm-tree-derived carbon used for electrode fabrication was examined by XRD, and the obtained pattern is presented in figure 1. The diffractogram exhibits two main structural features: a broad diffraction band associated with carbonaceous graphitic domains and several sharp reflections corresponding to crystalline potassium chloride. A broad diffraction band centred at approximately 2*θ* ≈ 24–25° is observed, which corresponds to the (0 0 2) reflection of turbostratic or partially ordered graphite [29]. The broad nature of this peak indicates limited long-range ordering of the graphene layers and reflects the presence of stacking disorder and structural defects, which are commonly observed in graphitized carbons derived from lignocellulosic biomass. Although the palm tree precursor was graphitized at elevated temperature, complete graphitic crystallinity is rarely achieved in biomass-derived carbons, resulting in broadened carbon reflections. The presence of this band confirms the formation of stacked sp^2^ carbon domains, indicating the successful carbonization and graphitization of the palm biomass.

**Figure 1.**
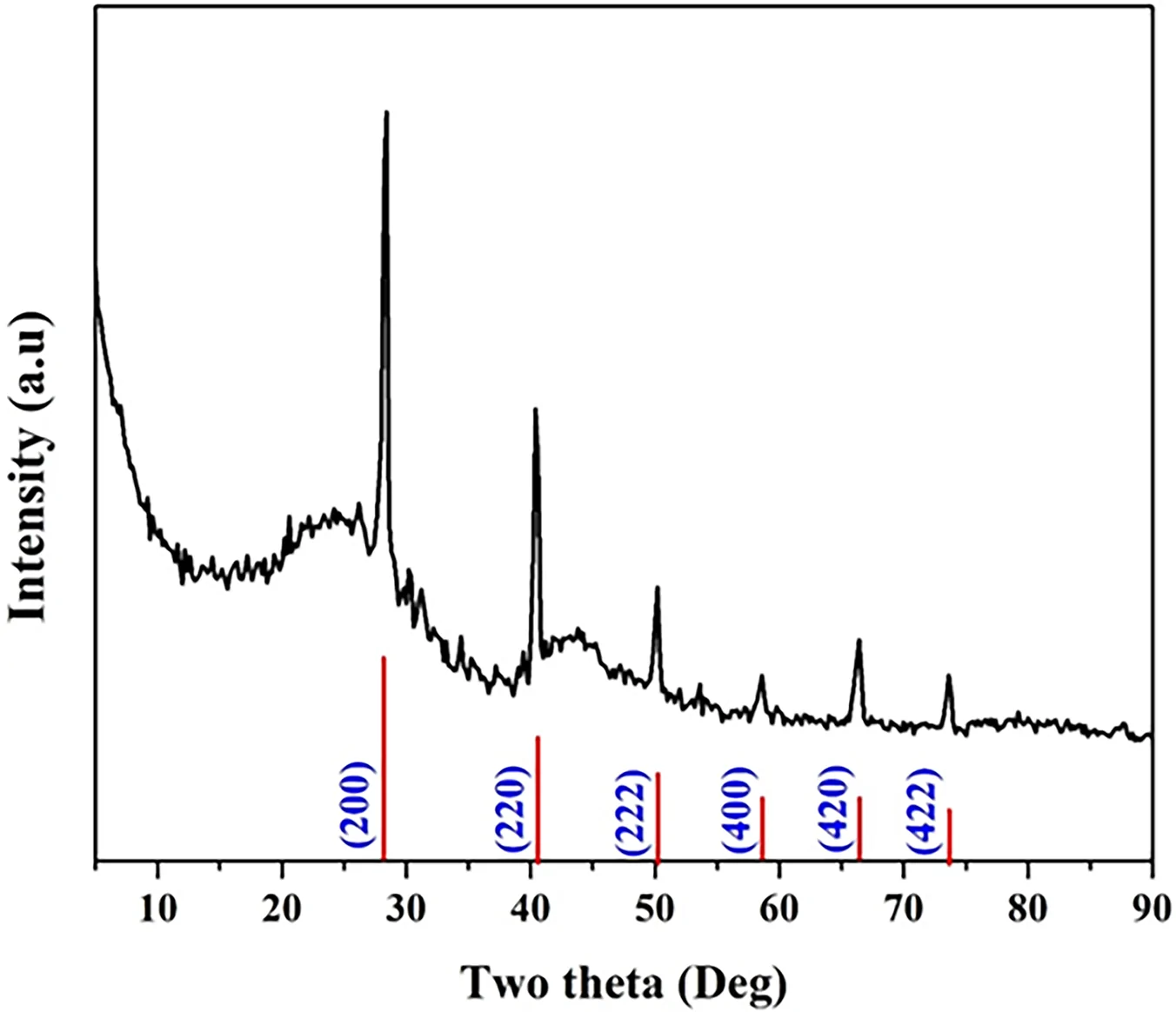
XRD analysis for the graphitized palm tree powder at 1000°C.

In addition to the graphitic carbon band, several sharp diffraction peaks appear at 2θ values of approximately 28.4, 40.4, 50.2, 58.6, 66.4 and 73.6°, which correspond to the (2 0 0), (2 2 0), (2 2 2), (4 0 0), (4 2 0) and (4 2 2) crystallographic planes of cubic potassium chloride, respectively. These reflections match well with the standard reference pattern (PDF #41-1476). The relatively high intensity and narrow width of these peaks indicate the presence of a well-crystallized inorganic phase with crystallite domains significantly larger than those of the surrounding carbon matrix. It is noteworthy mentioning that the XRD patterns for the three anodes, prepared at 900°C, 1000°C and 1100°C, were almost similar; figure 1 shows the corresponding pattern of 1000°C-graphitized palm tree. Further qualitative investigation of KCl in the graphitized biomass was carried out using AgNO_3_ aqueous solution (5 wt%). Briefly, the graphitized palm tree powder was immersed in a considerable amount of deionized water and stirred for 30 min, then the slurry was filtered. Drops of AgNO_3_ solution were added to the filtrate; the solution became cloudy owing to the formation of AgCl precipitate, as shown in the videoclip (supplementary material, S3).

The presence of crystalline KCl originates from the intrinsic mineral composition of palm biomass. Potassium is naturally abundant in plant tissues, and during the high-temperature graphitization process, potassium-containing organic species decompose and react with chlorine species present in the biomass matrix, forming thermodynamically stable potassium chloride. Although the graphitized palm tree was subsequently ground, mixed with starch and protamine binders, pressed into a moulded electrode and thermally treated, these processing steps mainly influence the morphology, mechanical integrity and electrical connectivity of the electrode, rather than altering its crystalline phase composition. Consequently, the dominant crystalline features of the material remain governed by the graphitic carbon framework and the mineral KCl phase inherited from the palm biomass.

#### 3.1.2. Surface morphology

The SEM images shown in figure 2A,B reveal that the prepared anode possesses a rough and heterogeneous surface composed of irregular, plate-like carbon fragments with different particle sizes. The carbon particles appear to be compactly assembled, yet they do not form a fully dense body. Instead, numerous voids and open spaces are clearly observed between the overlapped graphitic flakes, producing an apparently porous surface texture. At the lower magnification (figure 2A), these pores are seen as widely distributed interparticle cavities throughout the electrode surface, indicating that the moulded anode retained an open microstructure after pressing and post-thermal treatment. At the higher magnification (figure 2B), the pore network becomes more evident, where slit-shaped gaps and irregular channels can be distinguished between adjacent carbon sheets.

**Figure 2.**
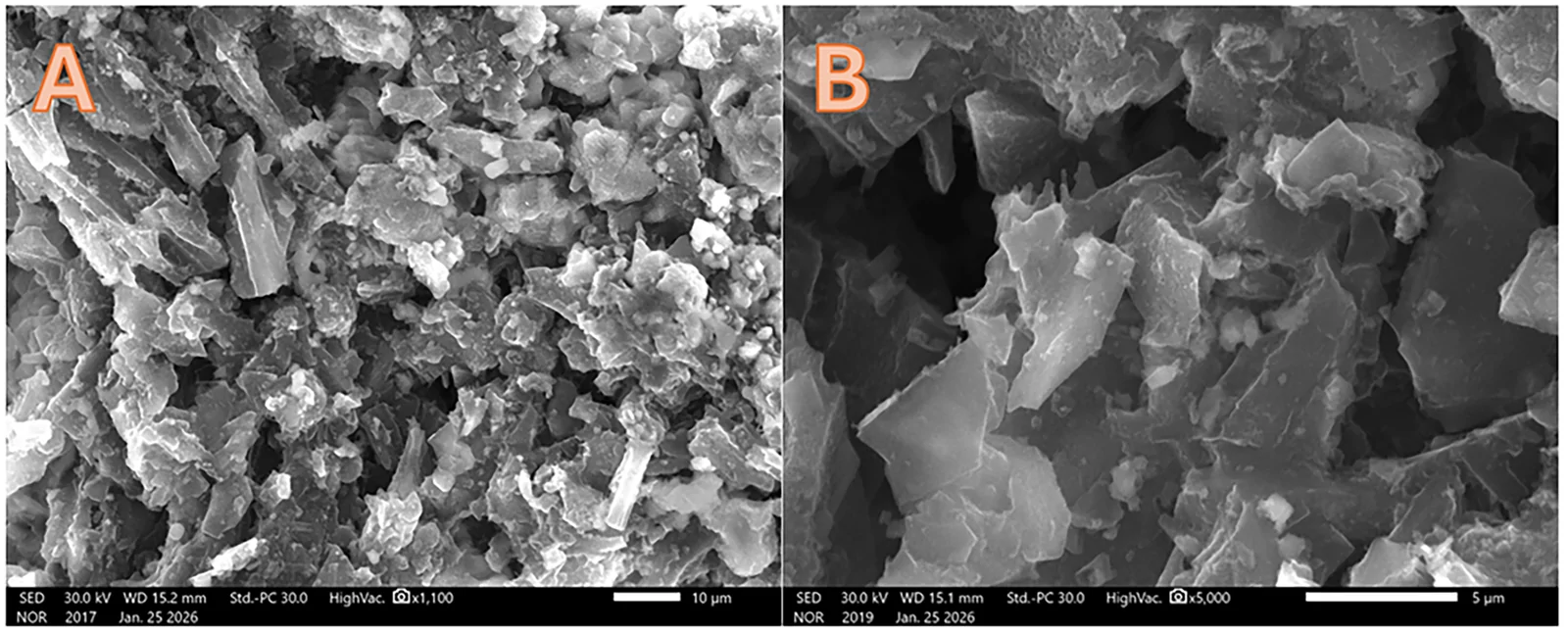
SEM images of the fabricated graphitized palm-tree-branch-based anode at different magnifications: (A) low-magnification image showing the overall rough and porous surface morphology and (B) high-magnification image revealing the flake-like graphitic structure and interparticle pores/channels distributed over the anode surface.

The observed porosity is highly favourable for MFC anode applications. First, the presence of open surface pores increases the accessible surface area available for microbial attachment and biofilm growth, which is essential for the establishment of an electroactive bacterial community [30]. Second, these pores can facilitate the penetration of wastewater into the interior region of the anode surface, thereby improving the contact between the electrode and the electrolyte [31]. Such an open structure is also beneficial for mass transport, as it allows easier diffusion of organic substrates, dissolved ions and metabolic intermediates through the electrode/electrolyte interface. In addition, the rough and porous morphology may create a larger number of active interfacial sites for extracellular electron transfer, which can contribute positively to the electrochemical performance of the anode.

#### 3.1.3. Electrochemical activity of the fabricated anodes

The cyclic voltammograms of the anodes prepared from palm tree branches graphitized at 900°C, 1000°C and 1100°C are presented in figure 3A–C, respectively. The measurements were conducted in the presence of real wastewater containing NaAc (6.25 g l^−1^). In general, all samples exhibit broad, featureless voltammetric responses without distinct anodic or cathodic redox peaks over the investigated potential window. This behaviour indicates that the fabricated anodes are predominantly carbonaceous in nature and are free from strongly electroactive surface species that could generate parasitic faradaic reactions. From the viewpoint of MFC application, this is an important observation because it suggests that the measured current is mainly associated with charge transfer through the conductive carbon framework and, during cell operation, with microbial electron harvesting rather than with redox-active impurities inherently present in the electrode material [32]. In other words, the proposed anode behaves as an electrochemically stable conductive substrate rather than a self-redox material that may interfere with the true bioelectrochemical response.

**Figure 3.**
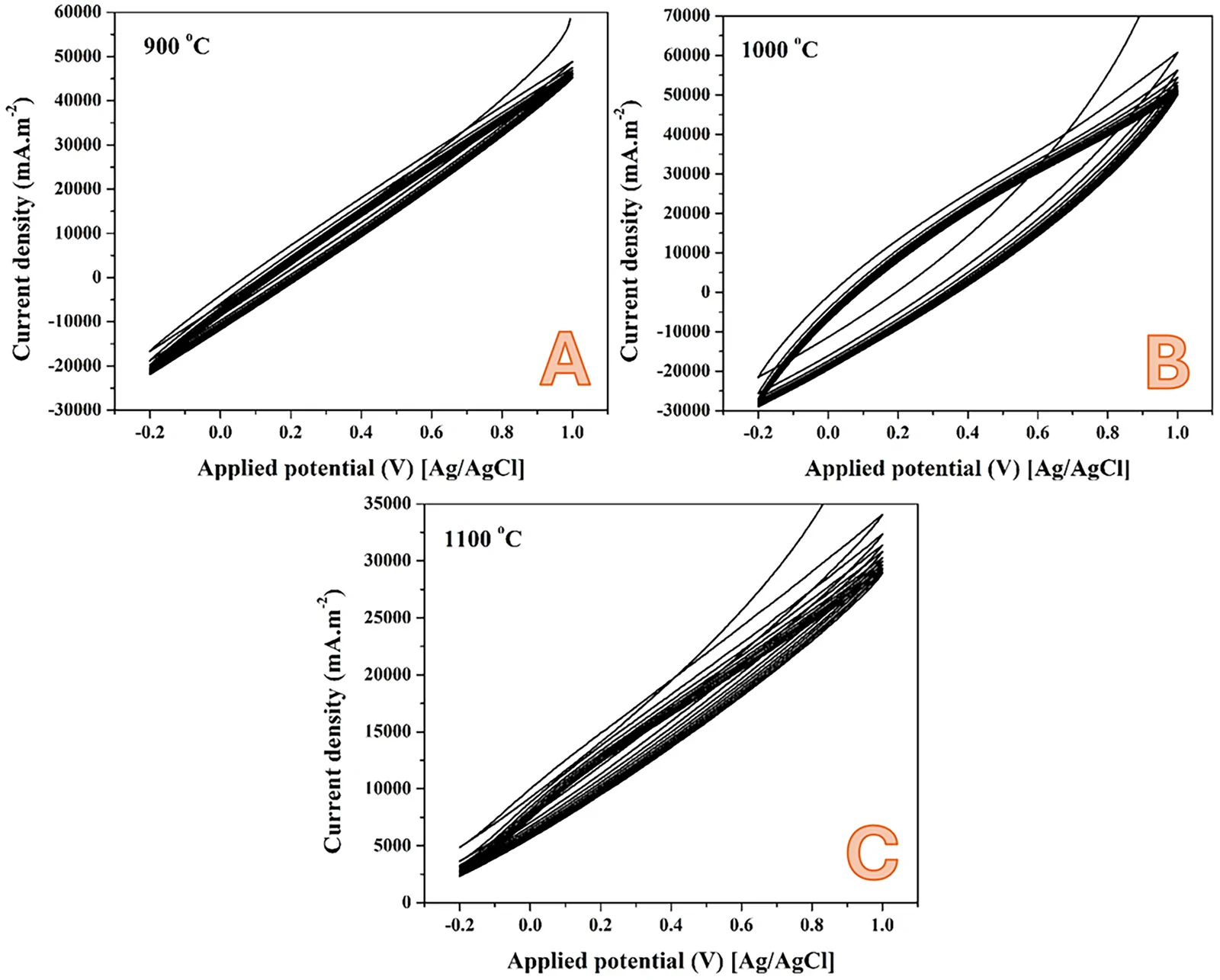
Cyclic voltammograms of the fabricated palm-tree-branch-based anodes prepared from materials graphitized at different temperatures: (A) 900°C, (B) 1000°C and (C) 1100°C. Measurements were performed in the assembled microbial fuel cell at a scan rate of 50 mV s^−1^ for 20 successive cycles.

Another important feature is the generally smooth shape of the voltammograms and the reasonably consistent overlap of the successive cycles, which indicates acceptable electrochemical stability of the moulded anodes under repeated polarization. This result confirms that the adopted fabrication strategy, involving powder consolidation with bitumen and starch followed by thermal treatment, produced electrodes with sufficient structural integrity to withstand repeated charging/discharging processes without obvious deterioration. The absence of abrupt current fluctuations or irregular peaks also suggests that the binder-derived carbonized framework did not introduce unstable electrochemical sites, which is favourable for long-term MFC operation [33].

Although no redox peaks are observed, the voltammogram shape still provides useful information regarding the electrochemical characteristics of the prepared anodes [34]. The responses are broad and enclosed, indicating the contribution of electrical double-layer charging and interfacial capacitive behaviour. Such capacitive characteristics are beneficial in MFC anodes because they can promote temporary charge storage at the electrode/electrolyte interface, smooth fluctuations in electron supply from the biofilm and facilitate more stable current output. Therefore, the CV results support the idea that the developed palm-derived anodes can provide not only an electrically conductive pathway but also an electrochemically accessible interface for charge accumulation and transfer.

A comparison among the three graphitization temperatures reveals clear differences in the magnitude and shape of the voltammetric loops. The anode prepared from the material graphitized at 900°C (figure 3A) shows a relatively narrow and nearly linear response, which suggests that the electrode behaviour is more strongly dominated by resistive conduction with comparatively limited electrochemically accessible surface area. This may be attributed to incomplete structural ordering of the carbon matrix at this temperature, where the conductivity is improved compared with raw biomass-derived carbon, but the graphitic network is still not fully developed. Consequently, although the 900°C anode is electrochemically active and stable, its interfacial charge-storage capability appears less pronounced than that of the anode prepared at 1000°C.

In contrast, the anode derived from the 1000°C graphitized material (figure 3B) displays the largest enclosed voltammetric area and the highest current response among the investigated samples. This broader loop indicates enhanced electrochemical accessibility, improved charge-transfer behaviour and a larger effective interfacial area. Such behaviour can reasonably be attributed to the attainment of a more favourable balance between graphitic ordering and structural openness at this temperature. The larger voltammetric area of this sample strongly suggests superior electrochemical activity, which is expected to translate into improved performance when the electrode is used as an MFC anode [35].

For the anode prepared from the material graphitized at 1100°C (figure 3C), the CV response remains smooth and stable but becomes less favourable in terms of overall current magnitude and loop development compared with the 1000°C sample. This result implies that increasing the graphitization temperature beyond 1000°C does not further enhance the electrochemical behaviour of the anode. A possible explanation is that excessive heat treatment may induce greater structural densification, sheet stacking or partial loss of electrochemically accessible surface features, thereby reducing the beneficial balance between conductivity and surface availability. Thus, although high-temperature graphitization can improve structural ordering, an overly severe treatment may reduce the number of accessible active sites and pore channels available for interfacial interaction with the electrolyte and microbial biofilm.

#### 3.1.4. Electrical conductivity of the fabricated anodes

The electrical conductivity behaviour of the prepared anodes is presented in figure 4, where the current response is plotted as a function of the applied potential for samples prepared from palm-tree-derived carbon graphitized at 900°C, 1000°C and 1100°C, in addition to the activated-carbon-based anode used for comparison. The measurement was conducted on the formulated anode before inserting in the MFC. A clear linear relationship between current and applied potential is observed for all samples, indicating that the prepared electrodes exhibit predominantly ohmic conduction behaviour within the investigated potential range. The absence of marked deviation from linearity also suggests that the fabricated anodes possess reasonably continuous conductive pathways and that no major electrical discontinuity was introduced during the moulding and post-thermal-treatment processes [36].

**Figure 4.**
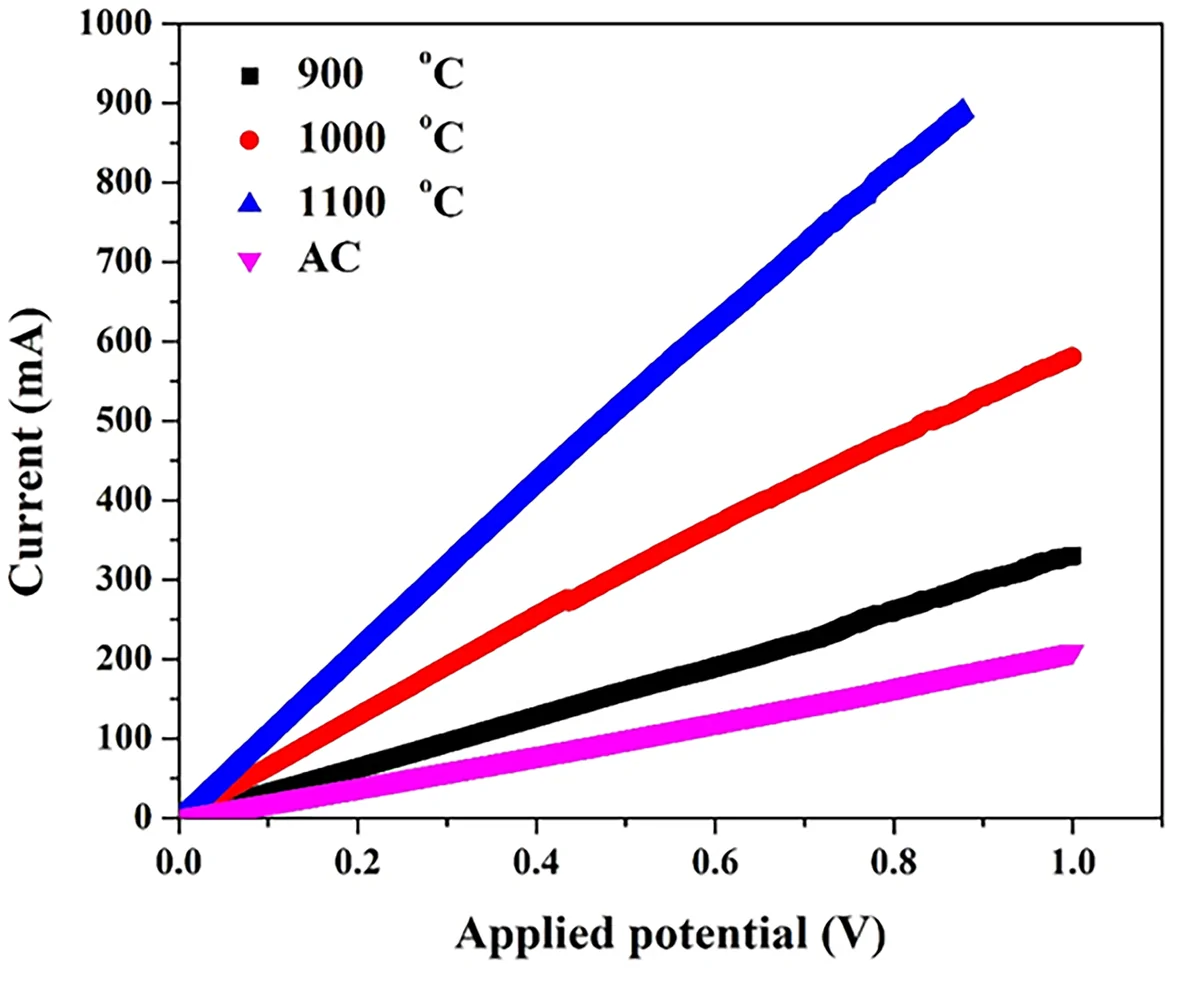
Current–potential curves of the fabricated anodes prepared from palm-tree-branch-derived carbon graphitized at different temperatures (900°C, 1000°C and 1100°C), compared with the activated-carbon-based anode (AC).

A pronounced effect of graphitization temperature on electrical conductivity can be clearly observed. The conductivity increases in the order: AC < 900°C < 1000°C < 1100°C. Among all tested samples, the anode prepared from palm tree branches graphitized at 1100°C displayed the highest current response at any given applied potential, confirming that it possesses the best electrical conductivity. The sample prepared at 1000°C showed intermediate behaviour, while the 900°C sample exhibited a lower conductivity, although it still outperformed the activated carbon anode. The enhancement in conductivity with increasing graphitization temperature can be attributed to progressive structural transformation of the biomass-derived carbon [37]. At higher thermal treatment temperatures, the carbon matrix generally undergoes a greater degree of aromatization and structural ordering, accompanied by the elimination of residual volatile species and the development of more continuous graphitic domains. These changes reduce electron-scattering sites and facilitate charge transport through the electrode body [38]. Another notable observation is that all palm-derived graphitized anodes exhibited substantially higher conductivity than the activated-carbon-based anode. This result confirms the advantage of converting palm tree branches into graphitized carbon rather than relying on conventional activated carbon as the primary anodic material.

### 3.2. Cell performance

#### 3.2.1. Comparison with AC-based anode

Figure 5 displays the performance of MFCs assembled using AC and graphitized palm trees at 1000°C-based anodes and derived from sewage containing 5 g l^−1^ sodium acetate electrolyte. The polarization and power-density data in figure 5A–D reveal a very large difference in MFC performance between the activated-carbon-based anode and the developed palm-tree-derived graphitized anode prepared at 1000°C. In the case of the AC anode, the cell initially exhibited very weak performance during the first 2 days, with an open-circuit voltage of only about 0.06–0.07 V, a short-circuit current density below 150 mA m^−2^ and a maximum power density of only about 2–3 mW m^−2^. This low initial response suggests that the AC-based anode did not provide a sufficiently favourable electrochemical interface for rapid establishment of an efficient electrogenic biofilm. As the operation continued, the cell performance gradually improved, reaching a maximum on approximately day 7, where the OCV increased to about 0.41 V, the current density reached about 415 mA m^−2^, and the power density attained approximately 52 mW m^−2^. This progressive increase is characteristic of biofilm maturation and indicates that the AC anode required a relatively long acclimatization period before reaching its optimum performance.

**Figure 5.**
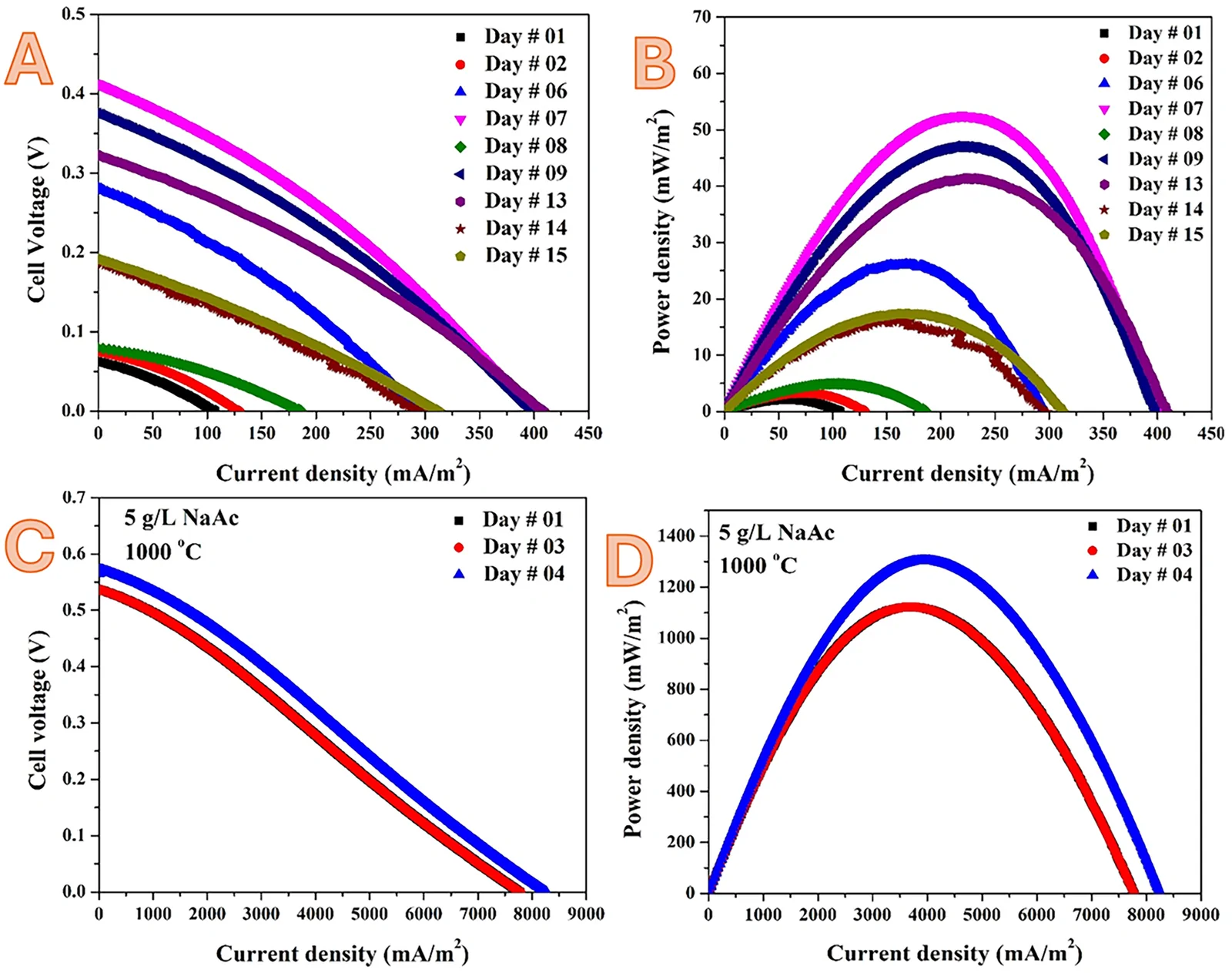
Performance comparison of microbial fuel cells employing the activated-carbon-based anode and the graphitized palm-tree-branch-based anode prepared at 1000°C. (A) Polarization curves of the MFC using the AC-based anode at different operation days; (B) corresponding power-density curves of the AC-based anode; (C) polarization curves of the MFC using the palm-derived graphitized anode at different operation days; and (D) corresponding power-density curves of the palm-derived graphitized anode. Both cells were driven by sewage wastewater containing 5 g l^−1^ NaAc.

After day 7, the AC-based cell exhibited a gradual decline in performance. Although the cell still maintained relatively high output on days 9 and 13, with maximum power densities of about 47 and 41 mW m^−2^, respectively, a substantial drop was observed by days 14 and 15, where the maximum power density decreased to about 15–17 mW m^−2^. This behaviour suggests that the AC anode suffered from limited operational stability under the investigated conditions. In any case, the AC anode showed not only low initial output but also a relatively narrow performance window centred around 1 optimum day.

A completely different behaviour was observed for the graphitized palm-tree-derived anode prepared at 1000°C, as shown in figure 5C,D. The polarization curves exhibit much higher open-circuit voltages and much broader current ranges than those obtained with the AC anode. The OCV was about 0.54–0.57 V, which is substantially higher than the best value recorded for the AC-based system. More importantly, the current density extended to approximately 7700–8200 mA m^−2^, which is nearly 20 times greater than that of the AC anode. Correspondingly, the maximum power density reached about 1120 mW m^−2^ on days 1 and 3 and further increased to nearly 1300 mW m^−2^ on day 4. This represents an enhancement of roughly 25-fold relative to the maximum value obtained with the AC anode.

The remarkable improvement in power generation strongly demonstrates the superiority of the proposed palm-derived graphitized anode over the conventional activated-carbon-based counterpart. Based on the best-performance condition in each case, the developed anode increased the OCV from about 0.41 to 0.57 V, the maximum current density from about 415 to 8200 mA m^−2^ and the maximum power density from about 52 to 1300 mW m^−2^. These improvements clearly confirm that the proposed anode is not merely a low-cost alternative but a substantially more effective anodic material for MFC operation.

Several structural and electrochemical features can explain this outstanding behaviour. First, the graphitized palm-derived anode possesses much higher intrinsic electrical conductivity than the AC-based electrode, as already demonstrated in the conductivity results. Second, the CV results indicated that the 1000°C graphitized sample exhibited the most favourable electrochemical response among the investigated palm-derived anodes, suggesting that this temperature provided an optimum balance between structural ordering and interfacial accessibility [39].

Unlike the AC-based system, which required about one week to reach its maximum output, the graphitized palm tree anode already delivered very high power within the first few days of operation. This behaviour suggests that the developed surface provided a more suitable environment for rapid development of an active electrogenic biofilm. The shorter activation period is a highly desirable feature for practical MFC applications, because it reduces startup time and improves process feasibility.

The shape of the polarization curves also provides useful insight. For the AC anode, the curves are confined to a narrow current range and exhibit relatively steep voltage decay, reflecting pronounced polarization losses. In contrast, the palm-derived anode maintains appreciable cell voltage over a much broader current-density range, indicating reduced polarization and improved overall electrochemical efficiency. From the approximate values extracted from the plots, the apparent area-specific internal resistance can be estimated by the ratio of OCV to short-circuit current density. On this basis, the AC anode gives an approximate value close to 1.0 Ω m^2^, whereas the developed palm-derived anode yields a much lower value near 0.07 Ω m^2^. Although this is only an approximate estimation from graphical data, it strongly supports the conclusion that the proposed anode significantly lowers the internal resistance of the MFC.

It should be noted that the different monitoring periods for the AC and 1000°C graphitized anodes reflect their different temporal performance evolution and were not designed as a matched long-term durability comparison. The graphitized anode developed a strong electrochemical response within the first few days, whereas the AC-based system exhibited a more gradual evolution over a longer period. Accordingly, the results demonstrate the rapid establishment and high short-term electrochemical performance of the 1000°C anode but should not be interpreted as evidence of superior long-term stability. Extended operation under identical monitoring periods will be required to establish comparative durability and long-term biofilm stability.

#### 3.2.2. Effect of graphitization temperature

The results in figure 6 clearly demonstrate that the graphitization temperature exerts a decisive influence on the performance of the proposed palm-tree-branch-based anode, even when the NaAc concentration is fixed at 7.5 g l^−1^. The most striking result is that the anode prepared from palm tree branches graphitized at 1000°C delivered the best overall performance. Its open-circuit voltage remained in the range of about 0.50–0.56 V, while the maximum current density extended to approximately 7600–8000 mA m^−2^ during the first 4 days of operation. The corresponding maximum power density reached about 1000 mW m^−2^ on day 1, increased to around 1110 mW m^−2^ on day 2 and attained its highest value of nearly 1160 mW m^−2^ on day 3. Even after some decline, the cell still maintained a relatively high-power density of about 850 mW m^−2^ on day 5. These results confirm that the 1000°C anode provided not only the highest power output but also a broad current-generating range and good operational persistence.

**Figure 6.**
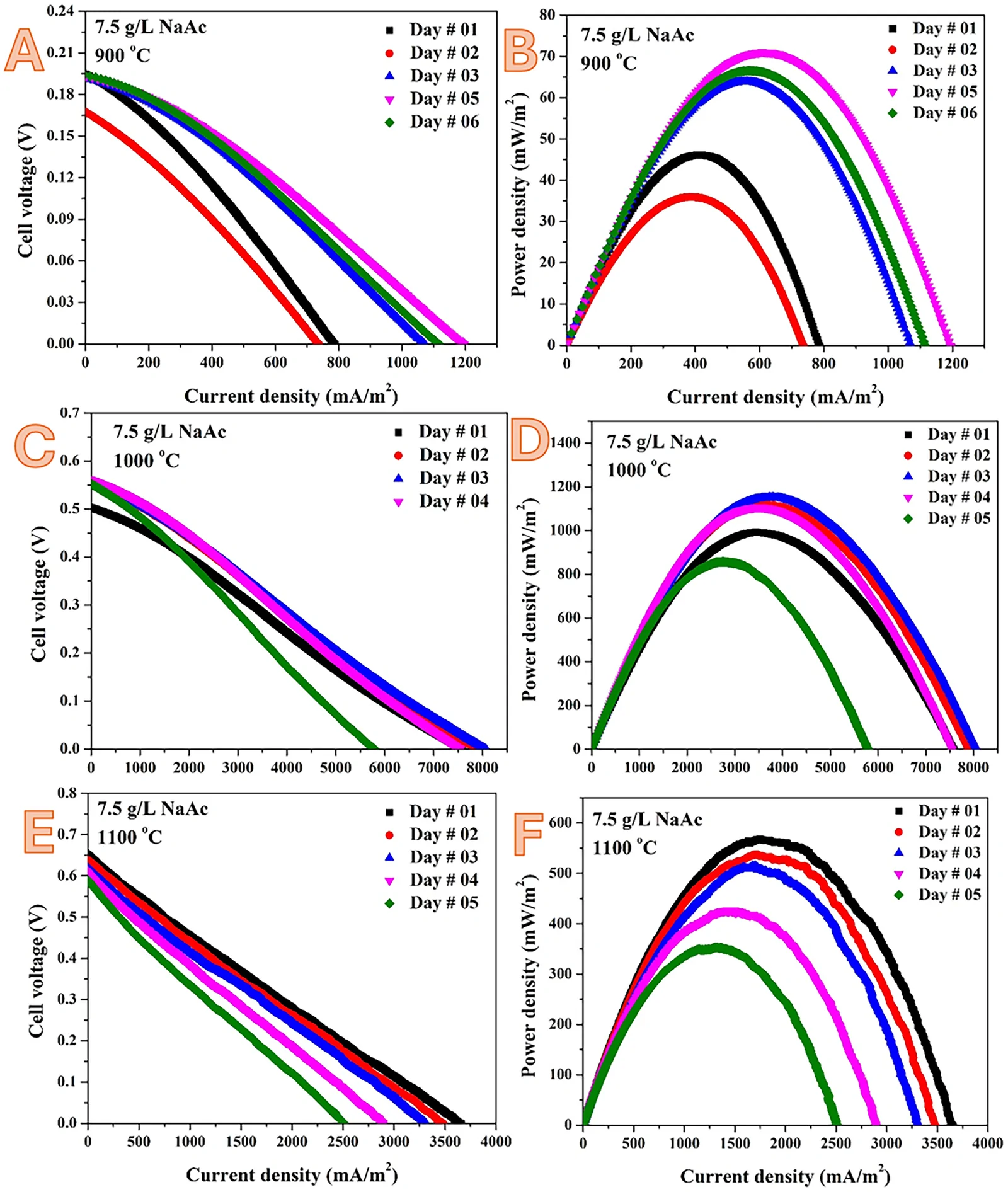
Effect of graphitization temperature on the performance of microbial fuel cells equipped with graphitized palm-tree-branch-based anodes at a fixed NaAc concentration of 7.5 g l^−1^. (A) Polarization curves of the MFC employing the 900°C anode; (B) corresponding power-density curves for the 900°C anode; (C) polarization curves of the MFC employing the 1000°C anode; (D) corresponding power-density curves for the 1000°C anode; (E) polarization curves of the MFC employing the 1100°C anode; and (F) corresponding power-density curves for the 1100°C anode.

By contrast, the 900°C anode exhibited much lower performance. Its OCV remained nearly constant at about 0.17–0.19 V, which is far below that of the other two graphitized samples. The maximum current density increased from about 730–800 mA m^−2^ during the first 2 days to about 1180–1200 mA m^−2^ on day 5, while the maximum power density rose from approximately 36–46 mW m^−2^ to a maximum of only about 71 mW m^−2^.

The 1100°C anode showed intermediate behaviour between the other two samples. Interestingly, it exhibited the highest OCV among all investigated anodes, starting at about 0.65 V on day 1 and gradually decreasing to about 0.58 V on day 5. It also produced relatively high-power output, with a maximum power density of about 565 mW m^−2^ on day 1. However, this value continuously decreased with operation time to about 350 mW m^−2^ by day 5. Similarly, the maximum current density declined from about 3700 mA m^−2^ to about 2500 mA m^−2^ over the same period. Thus, although the 1100°C anode performed much better than the 900°C sample, it remained distinctly inferior to the 1000°C anode in terms of both current density and maximum power density.

The overall performance order at 7.5 g l^−1^ NaAc can therefore be summarized as follows: 1000°C > 1100°C > 900°C. This trend is fully consistent with the electrochemical and physicochemical characteristics discussed earlier. The conductivity data showed that electrical conductivity increased with graphitization temperature and reached its highest value at 1100°C. However, the CV results revealed that the 1000°C sample exhibited the most favourable voltammetric behaviour, with the largest current response and the broadest loop area, indicating superior electrochemical accessibility.

The behaviour of the 1100°C anode is especially important because it shows that increasing conductivity alone does not guarantee the highest MFC performance. Despite having the highest conductivity and the highest OCV, this sample produced lower current density and lower power density than the 1000°C anode. A reasonable explanation is that excessive graphitization at 1100°C enhanced structural ordering but at the same time reduced surface accessibility, defect density or the abundance of active edge sites needed for efficient microbial interaction and extracellular electron transfer.

Another notable observation is the difference in day-to-day behaviour. The 900°C sample showed a gradual increase in performance up to day 5, followed by a slight decline on day 6, indicating a slower activation process. The 1000°C sample reached very high output rapidly and maintained it during the first several days, suggesting fast biofilm establishment and efficient anodic kinetics. In contrast, the 1100°C sample showed its best performance on day 1, followed by a progressive decline, which may indicate that although the initial electrochemical conditions were favourable, the anode surface was less suitable for sustaining long-term microbial activity at the same level. Therefore, in addition to achieving high peak power, the 1000°C anode also demonstrated the most desirable startup and operational stability characteristics.

The present time-dependent polarization measurements demonstrate the development and reproducibility of MFC performance under the investigated operating conditions; however, they should not be interpreted as a dedicated long-term durability assessment. Future work should therefore evaluate the developed anodes over extended and identical operating periods using continuous cell-voltage monitoring under a fixed external resistance or chronoamperometry at constant potential. Such measurements will enable quantitative assessment of long-term current stability, biofilm maturation, electrode durability and performance retention during prolonged sewage treatment.

#### 3.2.3. Performance of electrolyte-free MFCs

The most important result from figure 7 is that the three anodes, prepared from graphitized palm tree branches at different temperatures, were able to generate electricity even in the absence of externally added electrolyte (e.g. NaAc), confirming that the developed system can operate under electrolyte-free conditions using only sewage wastewater. Since XRD analysis confirmed the presence of KCl and the AgNO_3_ (videoclip in the supporting information file; supplementary material, figure S3) test verified chloride release from the anode, it is reasonable to conclude that the embedded salt contributed to the ionic environment around the anode and helped reduce ohmic limitations during cell operation. However, this contribution should be viewed as a supportive effect rather than a complete replacement for the bulk electrolyte phase.

**Figure 7.**
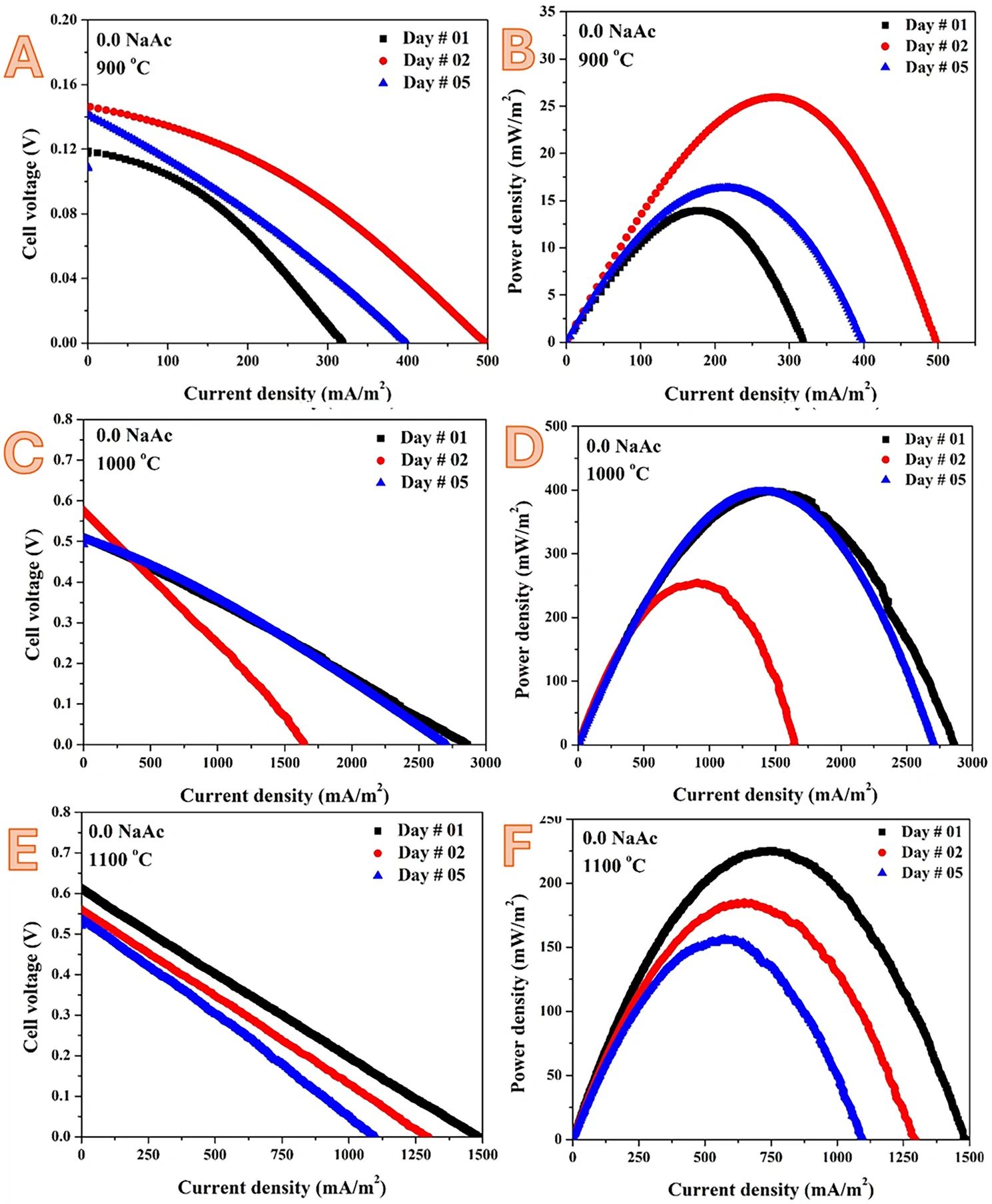
Polarization and power-density curves of electrolyte-free microbial fuel cells assembled with graphitized palm-tree-branch-based anodes prepared at different graphitization temperatures. (A) Polarization curves of the MFC employing the 900°C anode; (B) corresponding power-density curves for the 900°C anode; (C) polarization curves of the MFC employing the 1000°C anode; (D) corresponding power-density curves for the 1000°C anode; (E) polarization curves of the MFC employing the 1100°C anode; and (F) corresponding power-density curves for the 1100°C anode. All measurements were carried out in sewage wastewater without external NaAc addition.

A strong effect of graphitization temperature is again evident. The 900°C anode showed the weakest performance (figure 7A,B). Its OCV remained in the range of only 0.12–0.15 V, the maximum current density did not exceed about 490 mA m^−2^, and the maximum power density peaked at only about 26 mW m^−2^ on day 2. These low values suggest that graphitization at 900°C was insufficient to develop an adequately conductive and electrochemically accessible carbon network. This interpretation agrees well with the previously discussed conductivity data, where the 900°C sample displayed the lowest conductivity among the palm-derived graphitized anodes, and with the CV results, where it showed a narrower voltammetric response than the 1000°C sample.

Among the tested samples, the 1000°C anode exhibited the best overall performance under electrolyte-free conditions (figure 7C,D). It delivered a maximum power density of about 400 mW m^−2^ on both day 1 and day 5, with a corresponding maximum current density of about 2700–2900 mA m^−2^ and an OCV around 0.50–0.57 V. These values are markedly higher than those obtained with the 900 and 1100°C anodes, showing that the 1000°C material provided the most suitable balance of properties for practical MFC operation in untreated sewage [40].

The 1100°C anode exhibited intermediate behaviour (figure 7E,F). On day 1, it showed the highest OCV among all tested samples, about 0.62 V, and a maximum power density of approximately 225 mW m^−2^, but this performance gradually declined to about 155 mW m^−2^ by day 5. This result is particularly informative when considered together with the conductivity measurements. Although the 1100°C sample had the highest electrical conductivity, it did not provide the highest MFC output. This confirms that electrical conductivity alone does not determine anode performance in MFCs. In addition to charge transport, the electrode must provide an electrochemically accessible surface, suitable pore architecture, and a favourable interface for microbial attachment and extracellular electron transfer. Excessive graphitization may have increased structural ordering at the expense of surface accessibility, edge defects or biofilm-supporting features.

The day-to-day variation in figure 7 also provides useful insight into the role of retained KCl and the overall stability of the anodes. For the 900°C sample, the power density increased from about 14 mW m^−2^ on day 1 to 26 mW m^−2^ on day 2, then decreased to about 16–17 mW m^−2^ on day 5. This suggests an initial activation period followed by rapid decline, possibly owing to limited structural/electrochemical suitability of the anode and depletion of the initial ionic or substrate advantage. For the 1100°C anode, the gradual decrease from day 1 to day 5 may indicate that the initial benefit associated with high conductivity and/or salt release was not matched by long-term interfacial stability or sustained biofilm development. By contrast, the 1000°C sample showed high power from the beginning and retained nearly the same maximum output by day 5, which is a strong indication of rapid startup and comparatively stable operation.

It should be noted that ‘electrolyte-free’ operation in this study refers to the absence of externally added supporting electrolyte; the sewage wastewater inherently contains dissolved ionic species, while the graphitized palm-derived anode can additionally release intrinsic KCl into the medium.

#### 3.2.4. Effect of NaAc content

The results in figure 8 clearly show that the concentration of sodium acetate has a pronounced effect on the electrochemical performance of the MFC employing the 1100°C anode. In general, increasing the NaAc concentration from 5.0 to 6.25 g l^−1^ led to a substantial improvement in both current generation and power output, whereas a further increase to 7.5 g l^−1^ caused a noticeable decline. This behaviour indicates that the influence of NaAc is not monotonic and that an optimum concentration exists for this anode. Among the investigated conditions, the best overall performance was obtained at 6.25 g l^−1^ NaAc.

**Figure 8.**
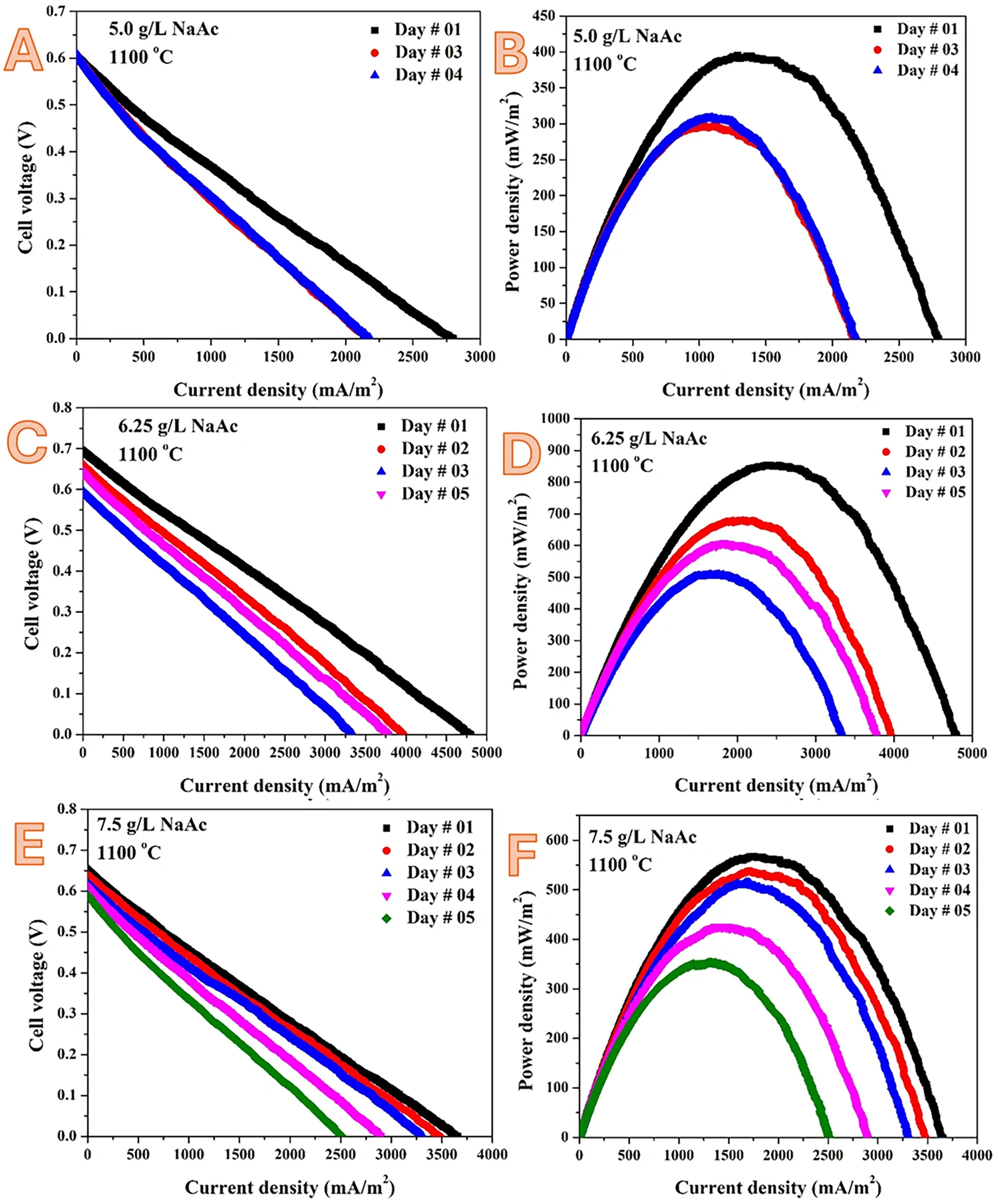
Effect of sodium acetate concentration on the performance of microbial fuel cells equipped with the graphitized palm-tree-branch-based anode prepared at 1100°C. (A) Polarization curves at 5.0 g l^−1^ NaAc; (B) corresponding power-density curves at 5.0 g l^−1^ NaAc; (C) polarization curves at 6.25 g l^−1^ NaAc; (D) corresponding power-density curves at 6.25 g l^−1^ NaAc; (E) polarization curves at 7.5 g l^−1^ NaAc; and (F) corresponding power-density curves at 7.5 g l^−1^ NaAc.

At 5.0 g l^−1^ NaAc, the MFC showed the lowest performance among the three tested acetate concentrations. The open-circuit voltage was around 0.60 V, the maximum current density reached only about 2100–2800 mA m^−2^, and the highest power density was approximately 395 mW m^−2^ on day 1, decreasing to around 300–310 mW m^−2^ on days 3 and 4. These results suggest that, although the 1100°C anode was capable of generating measurable electricity at this acetate level, the amount of readily biodegradable substrate and the resulting electrochemical activity were not sufficient to fully exploit the potential of the electrode. In other words, the substrate supply under this condition appeared to be suboptimal for maximizing the electron flux towards the anode.

A remarkable enhancement was observed when the NaAc concentration was increased to 6.25 g l^−1^. Under this condition, the cell exhibited the highest OCV, reaching about 0.70 V on day 1, together with the highest maximum current density of nearly 4750 mA m^−2^ and the highest maximum power density of about 850 mW m^−2^. Even after some decrease during subsequent days, the performance remained superior to that obtained at both 5.0 and 7.5 g l^−1^. For instance, the maximum power density was still about 680 mW m^−2^ on day 2 and recovered to approximately 600 mW m^−2^ on day 5. These values demonstrate that 6.25 g l^−1^ NaAc provided the most favourable balance between substrate availability and electrochemical stability for the 1100°C anode.

When the NaAc concentration was further increased to 7.5 g l^−1^, the performance deteriorated relative to the optimum value at 6.25 g l^−1^. Although the MFC still displayed better output than that obtained at 5.0 g l^−1^, the maximum power density decreased to about 565 mW m^−2^ on day 1 and gradually dropped to about 350 mW m^−2^ by day 5. Similarly, the maximum current density decreased from about 3700 mA m^−2^ on day 1 to about 2500 mA m^−2^ on day 5. Therefore, increasing the acetate concentration beyond 6.25 g l^−1^ did not further enhance the cell output and instead imposed a negative effect on both peak performance and operational persistence.

The overall trend can thus be summarized as follows: 6.25 > 7.5 > 5.0 g l^−1^ for the 1100°C graphitized palm-tree-based anode. This result is scientifically important because it confirms that NaAc does not act merely as a simple electrolyte additive [16]. Rather, it affects the MFC through multiple coupled mechanisms, including improvement of ionic strength, enhancement of substrate availability for electrogenic microorganisms and modulation of the interfacial bioelectrochemical environment. At low acetate concentration (5.0 g l^−1^), the system appears to suffer from insufficient substrate/electron-donor availability, leading to limited current generation. At the optimum concentration (6.25 g l^−1^), the microbial community likely received an adequate and balanced carbon source that promoted efficient oxidation and electron transfer without imposing excessive metabolic or transport limitations. At the higher concentration (7.5 g l^−1^), however, the excess acetate may have caused unfavourable effects such as local accumulation of intermediate metabolites, enhanced concentration polarization, altered pH conditions near the biofilm/electrode interface or biofilm overgrowth that hindered effective charge transfer.

Another notable observation is the day-to-day behaviour at the three acetate levels. At 5.0 g l^−1^, the best performance was obtained on the first day, followed by a decline, indicating that the system could not sustain a high bioelectrochemical activity for long under limited acetate availability. At 7.5 g l^−1^, the cell also showed progressive deterioration with operation time, suggesting that the initially available excess substrate did not translate into long-term stability and may have even accelerated performance loss. In contrast, the 6.25 g l^−1^ condition not only produced the highest initial output but also maintained comparatively high performance over several days, with a slight recovery on day 5 relative to day 3. This suggests that the intermediate acetate concentration created the most favourable environment for both startup and sustained electrogenic activity.

A comparison between the best-performance conditions provides a clearer perspective. Increasing NaAc from 5.0 to 6.25 g l^−1^ enhanced the maximum power density from about 395 to 850 mW m^−2^, corresponding to an increase of roughly 2.2 times. However, further increasing the concentration to 7.5 g l^−1^ reduced the maximum power density to about 565 mW m^−1^, which is approximately 34% lower than the optimum value at 6.25 g l^−1^. A similar trend is observed for the maximum current density, which increased from about 2800 mA m^−2^ at 5.0 g l^−1^ to about 4750 mA m^−2^ at 6.25 g l^−1^ and then decreased to about 3700 mA m^−2^ at 7.5 g l^−1^. These quantitative changes strongly confirm the existence of an optimum acetate concentration for this anode.

#### 3.2.5. Response surface analysis of NaAc-assisted MFC performance

To further interpret the combined effects of NaAc concentration, graphitization temperature and working days on the MFC performance, the experimental data were analysed using Design-Expert based on a quadratic response surface methodology (RSM) model. In the developed coded models, *A* represents the NaAc content, *B* the graphitization temperature and *C* the working day. The generated plots in figure 9 are standard RSM outputs: panels (A,C,E) are two-dimensional contour plots, while panels (B,D,F) are the corresponding three-dimensional response surface plots. These figures illustrate the interactive effect of NaAc content and graphitization temperature, while the third factor, working days, is held at its centre level by the software.

OCP=0.5520−0.0229A+0.2187B−0.0018C−0.0018AB+0.0016AC(3.1)−0.0250BC+0.0030A2−0.1681B2−0.0133C2CD=6368.61+1155.41A+775.71B−147.09C+434.23AB−264.48AC(3.2)−400.19BC−750.33A2−4553.38B2−141.67C2PD=938.41+165.75A+141.22B−27.53C+85.61AB−45.52AC(3.3)−61.77BC−128.20A2−688.85B2−14.45C2

**Figure 9.**
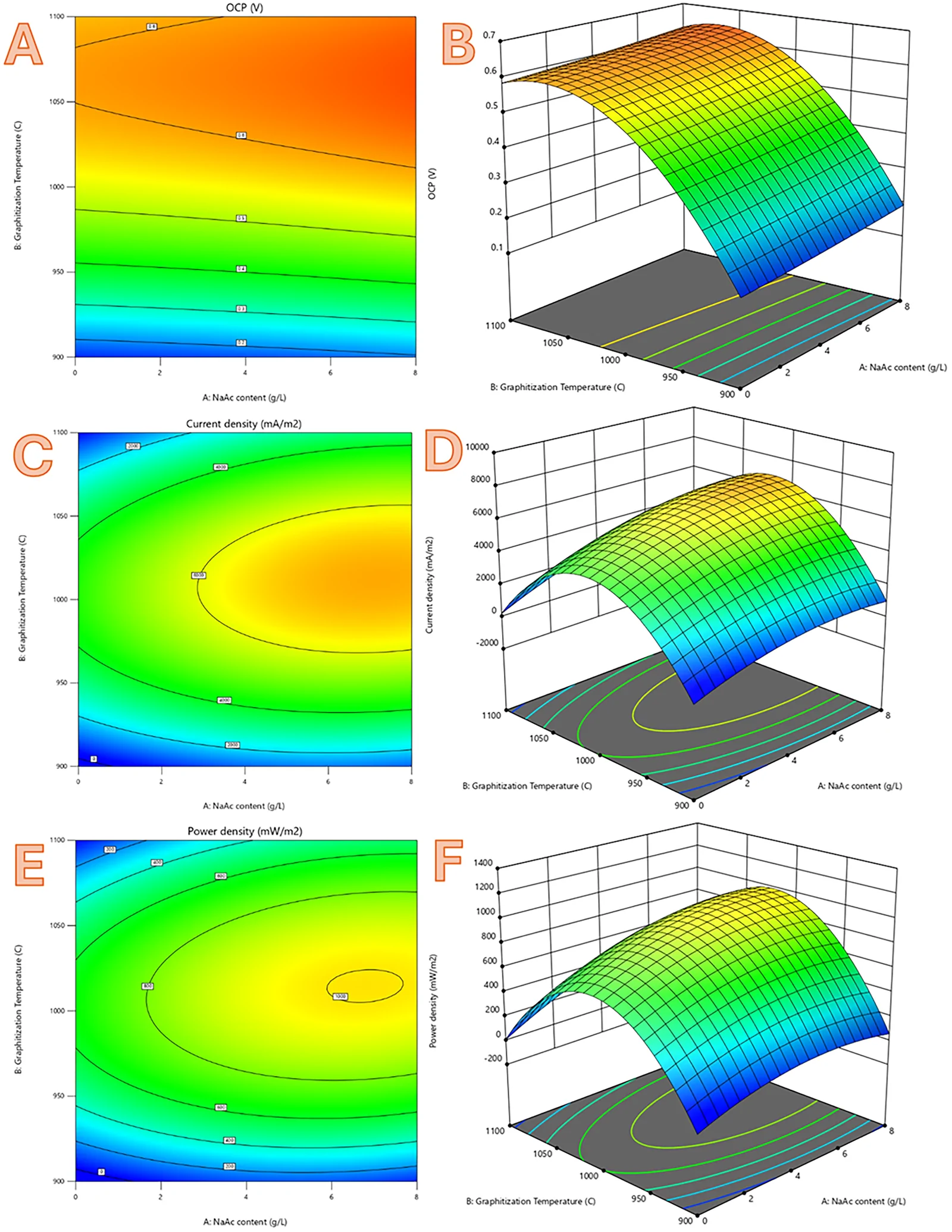
Response surface methodology (RSM) plots generated by Design-Expert showing the combined effect of NaAc concentration and graphitization temperature on the microbial fuel cell performance, with working days held at the centre level: (A) contour plot of open-circuit potential (OCP), (B) corresponding 3D response surface for OCP, (C) contour plot of current density, (D) corresponding 3D response surface for current density, (E) contour plot of power density, and (F) corresponding 3D response surface for power density.

The ANOVA results showed that the three models were statistically significant, with *p* < 0.0001, confirming that the selected factors exerted a real effect on the investigated responses. The model developed for OCP exhibited excellent agreement with the experimental data, with *R²* = 0.9868, whereas the models for current density and power density showed good predictive ability, with *R²* = 0.8615 and 0.8459, respectively. However, the significant lack of fit obtained for all models (*p* < 0.0001) indicates that the quadratic equations do not fully capture all of the experimental variability, most likely because the MFC system includes strong biological and time-dependent fluctuations. Accordingly, these models are best interpreted as tools for trend analysis and approximate optimization, rather than exact predictive equations.

The contour and surface plots for OCP (figure 9A,B) show that the open-circuit potential is affected mainly by the graphitization temperature, whereas the influence of NaAc content is comparatively smaller. This is evident from the nearly horizontal contour lines, which indicate that moving along the temperature axis causes a much larger change in OCP than moving along the NaAc axis. In general, OCP increases markedly with increasing graphitization temperature, and the highest values are located near the upper temperature boundary of the investigated domain. This behaviour is fully consistent with the experimental polarization results, where the 1100°C anode frequently displayed the highest OCV values. Such behaviour can be attributed to the progressive increase in structural ordering and electrical continuity of the carbon matrix as the graphitization temperature rises. The NaAc content shows only a weak curved effect on OCP, indicating that acetate concentration is not the primary factor controlling the thermodynamic cell voltage.

In contrast, the response surfaces for current density (figure 9C,D) and power density (figure 9E,F) exhibit closed elliptical contours, which are characteristic of a true optimum existing experimental domain. Unlike OCP, both current density and power density are governed by a stronger interaction between NaAc concentration and graphitization temperature. The responses initially increase with increasing NaAc content and graphitization temperature and then decline when either factor becomes excessively high. This creates a dome-shaped three-dimensional surface, indicating that the best MFC performance is obtained not at the extreme conditions, but at an intermediate optimum.

For current density, the model predicts that the highest values are located approximately around 1000–1020°C and 6–7 g l^−1^ NaAc, which agrees well with the experimental findings. At lower graphitization temperature, especially 900°C, the current response remains weak because the carbon matrix is insufficiently developed in terms of conductivity and interfacial electrochemical activity. On the other hand, although the 1100°C sample showed the highest electrical conductivity and often higher OCV, its current density did not reach the maximum values recorded for the 1000°C sample. This confirms that the MFC current response is not controlled by conductivity alone, but by the balance between conductivity, surface accessibility, pore architecture and suitability for microbial colonization.

A similar trend is observed for power density, which is the most practically relevant response. The power-density contour plot and the associated 3D response surface indicate that the maximum predicted output lies near 1000–1020°C and approximately 6–6.5 g l^−1^ NaAc. This result is highly meaningful because it explains the trend observed experimentally: although graphitization at 1100°C improves the electrical conductivity and OCP, it does not produce the highest power density. Instead, the optimum lies at a slightly lower graphitization temperature, where the anode appears to retain a more favourable balance between graphitic conductivity and interfacial bioelectrochemical properties. The model therefore supports the conclusion drawn from SEM, CV and conductivity measurements that the 1000°C anode provides the most suitable compromise between structural ordering and surface functionality.

The mathematical form of the models also supports this interpretation. In both the current-density and power-density equations, the linear terms of *A* and *B* are positive, showing that increasing NaAc content and graphitization temperature initially enhances performance. However, the quadratic terms *A*^2^ and *B*^2^ are negative, confirming the existence of a maximum beyond which further increase becomes unfavourable. In addition, the positive *AB* interaction term indicates that the beneficial effect of NaAc becomes more pronounced when it is combined with an appropriate graphitization temperature. This synergistic effect is clearly reflected by the elliptical contour plots.

Another important outcome from the models is the effect of working days. In all three equations, the linear coefficient of *C* is negative, and the quadratic *C*^2^ term is also negative. This indicates that prolonged operation generally leads to a reduction in OCP, current density and power density after the optimum period is reached. This trend agrees with the experimentally observed day-to-day behaviour, where the MFCs often reached their highest output during the early or intermediate days of operation, followed by gradual decline. Such deterioration may be related to substrate depletion, accumulation of metabolites, increased internal resistance or changes in biofilm structure with time.

#### 3.2.6. Surface morphology of the used anode

The SEM images of the used anode surface (1000°C anode with NaAc solution; figure 10A,B) reveal a pronounced morphological change compared with the unused anode discussed in figure 1. In the fresh electrode, the surface was characterized by exposed graphitic flakes, open interparticle voids and a rough porous texture, which provided a large accessible area for contact with the wastewater medium. After MFC operation, however, this originally open carbonaceous surface became largely covered by a newly formed layer composed of dense irregular deposits and abundant elongated rod- or needle-like features distributed over the electrode surface. The appearance of these aggregates strongly suggests the development of a biological coating on the anode during cell operation.

**Figure 10.**
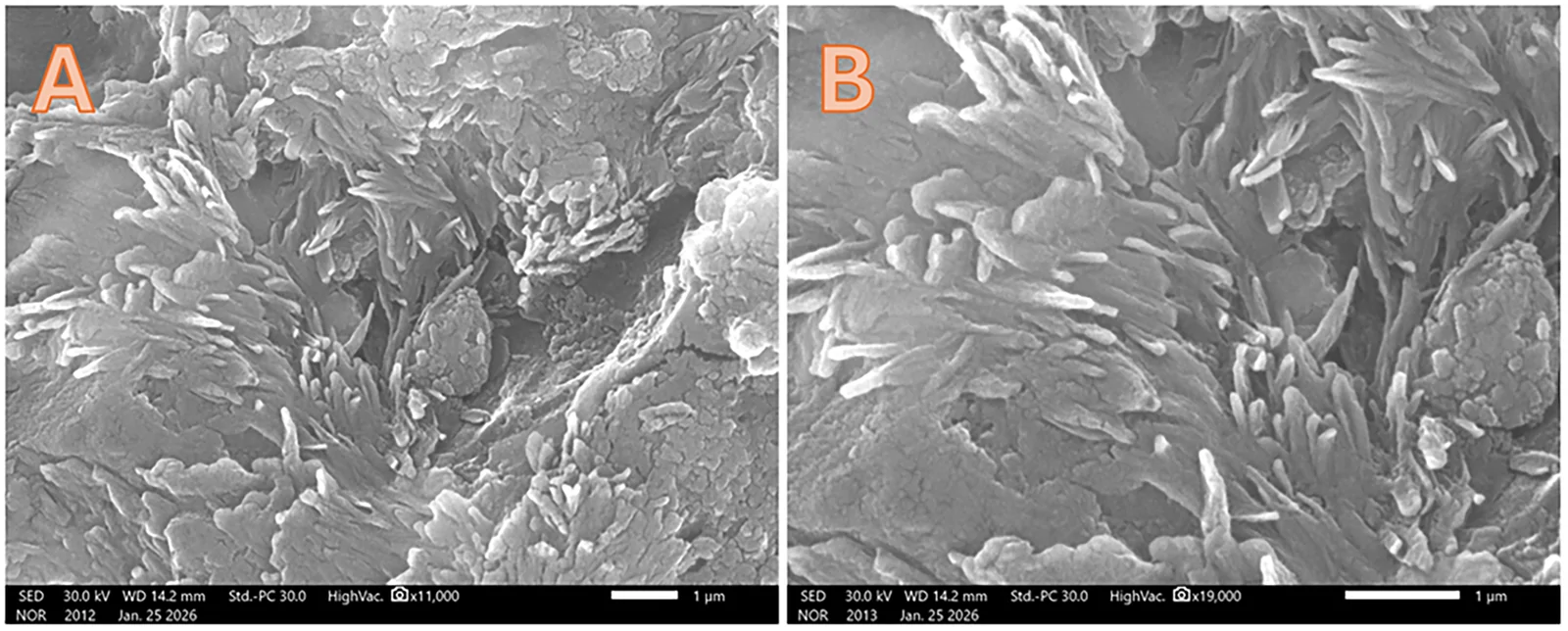
SEM images of the used graphitized palm-tree-branch-based anode operated under the optimum condition (1000°C, 5 g l^−1^ NaAc) at two magnifications: (A) lower-magnification image showing extensive surface coverage by a dense biofilm-like layer and (B) higher-magnification image revealing clustered rodlike microbial features and extracellular deposits formed on the anode surface after MFC operation.

Compared with the bare anode, where the graphitic plates and pores were clearly visible, the used anode exhibits partial masking of these surface features by the deposited layer. This observation indicates that the surface was actively colonized during operation and that the initially available rough and porous structure served as an effective platform for microbial attachment. The elongated clustered features observed in figure 10A and more clearly in figure 10B are consistent with biofilm-associated structures formed by microorganisms and their extracellular polymeric matrix. Although SEM alone cannot provide taxonomic identification of the attached species, the morphology strongly supports the formation of an electroactive biofilm on the anode surface.

This result is highly meaningful because it directly relates the earlier morphology of the fresh anode to its electrochemical performance in the MFC. The unused anode exhibited a rough, flake-like and porous structure that was considered advantageous for microbial adhesion, electrolyte penetration and mass transfer. After operation, the SEM images confirm that this surface indeed promoted extensive colonization, indicating that the proposed palm-derived graphitized anode provided a favourable interface for microbial growth. The porous architecture likely facilitated retention of microorganisms inside surface cavities, while the rough graphitic domains offered anchoring sites for stable biofilm establishment.

Another important point is that the biofilm-like layer appears well integrated with the carbon substrate rather than loosely deposited on a smooth inert surface. This suggests good compatibility between the prepared anode and the indigenous microbial community present in the sewage wastewater. Such compatibility is essential for sustained extracellular electron transfer, because intimate contact between microbial cells and the conductive surface is required for efficient harvesting of biologically generated electrons. Therefore, the used-electrode morphology provides direct visual support for the excellent performance observed with the 1000°C, 5 g l^−1^ NaAc condition.

The marked difference between the unused and used anodes also helps explain why the 1000°C sample outperformed the other graphitization temperatures. Earlier results showed that this anode offered the best balance between conductivity, accessible porosity and electrochemical activity. The present SEM images indicate that these properties were also highly favourable for microbial colonization, allowing the formation of a dense surface-associated layer during operation. In this respect, the superior MFC performance of the 1000°C anode can be attributed not only to its electrical properties but also to its strong ability to support biofilm development.

#### 3.2.7. Electrochemical impedance spectroscopy analysis

EIS was performed for the MFC equipped with the 1000°C graphitized palm-tree-based anode in the presence of 5 g l^−1^ NaAc, which was previously identified as the optimum operating condition. The Nyquist plots for the whole cell and the anode are shown in figure 11A,B, respectively. In both cases, the spectra are composed of a small depressed high-frequency semicircle followed by an inclined low-frequency tail, indicating that the cell impedance is governed by a combination of ohmic resistance, interfacial charge-transfer processes and diffusion/mass-transfer limitations. The high-frequency intercept on the real axis corresponds to the ohmic resistance (*R_s_*), which includes the contributions of the electrolyte, electrode body, current collectors and contact resistances. The whole-cell spectrum exhibited an *Rs* value of about 33–34 Ω, whereas the isolated anode showed a slightly lower value of about 27–28 Ω. This difference indicates that the additional resistance of the complete MFC mainly arises from the cathode side, the bulk electrolyte path and the overall reactor assembly. The relatively low *R_s_* values are consistent with the good conductivity of the proposed anode and with the favourable ionic environment provided by the NaAc-containing wastewater.

**Figure 11.**
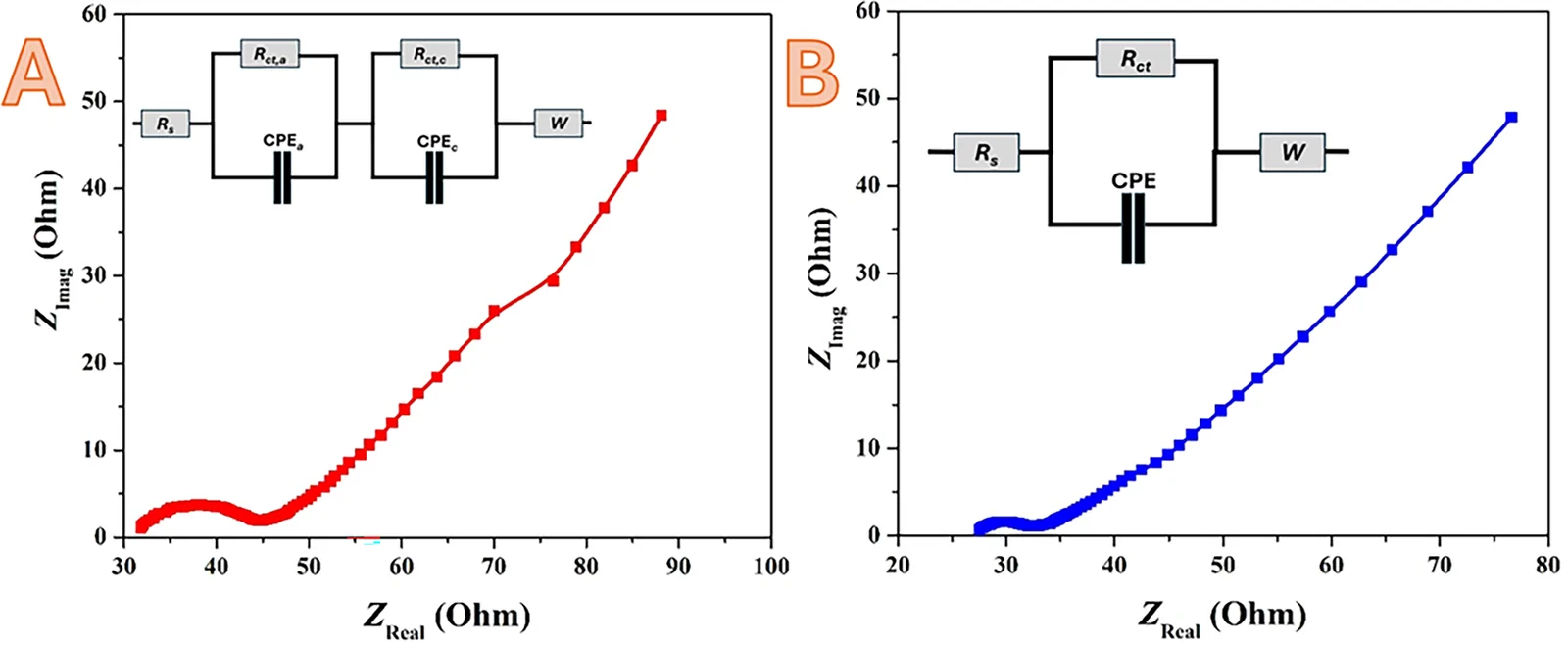
Nyquist plots obtained for the microbial fuel cell employing the graphitized palm-tree-branch-based anode prepared at 1000°C in the presence of 5 g l^−1^ NaAc: (A) impedance spectrum of the whole cell and (B) impedance spectrum of the anode. The inset in each panel shows the equivalent circuit used to interpret the corresponding EIS response.

A small depressed semicircle is observed in the high-frequency region for both spectra. This semicircle is associated with the interfacial charge-transfer resistance together with nonideal double-layer capacitance. The semicircle diameter is clearly larger for the whole cell than for the isolated anode, giving an approximate interfacial resistance of about 12–14 Ω for the whole cell and only 7–8 Ω for the anode. This result suggests that the anodic electron-transfer process is relatively facile and that a significant part of the additional interfacial loss in the whole-cell measurement originates from the cathode reaction and/or the overall cell architecture [41,42]. In other words, the proposed anode provides a comparatively efficient interface for bioelectrochemical charge transfer, which is fully consistent with its superior MFC performance discussed earlier.

Another important observation is that the semicircle is depressed rather than ideal, indicating nonideal capacitive behaviour. This is expected in porous bioelectrochemical systems, where the electrode surface is heterogeneous and covered by a biofilm. The nonideal response can be attributed to the rough graphitic surface, the porous structure of the moulded anode, and the distribution of active sites and time constants across the electrode/electrolyte interface. This interpretation is also consistent with the SEM observations, which showed a rough porous fresh anode and a biofilm-like layer after operation.

At lower frequencies, both spectra change into a long-inclined tail, which is characteristic of diffusion-controlled and mass-transfer processes. This region is commonly attributed to ion transport through the electrolyte and porous electrode structure, diffusion of ionic species within the biofilm/electrode interface and transport limitations associated with substrate and reaction products [43]. The low-frequency tail is more extended than the semicircle region, indicating that mass transport constitutes a major contribution to the total impedance under the optimum operating condition [44]. Thus, although the 1000°C anode exhibits low ohmic and charge-transfer resistance, the MFC performance is still influenced by diffusion-related limitations at low frequency.

A comparison between the two spectra provides additional insight into the source of the internal resistance. The whole-cell plot is shifted to higher *Z*_real_ values compared with the anode plot, but the two spectra exhibit similar overall shapes. This indicates that the same fundamental processes govern the impedance response in both cases, while the whole cell includes extra contributions from the cathode and the complete reactor configuration. Since the anode spectrum already spans a large portion of the total impedance window, it can be concluded that the anode accounts for a substantial fraction of the total cell resistance. However, the additional shift in the whole-cell spectrum confirms that cathodic polarization and cell-assembly losses are not negligible.

The inset equivalent circuits shown in figure 11A,B were selected to represent the main physicochemical processes governing the impedance response of the studied MFC system. For the whole cell (figure 11A), the circuit consists of the solution/ohmic resistance (*R_s_*), followed by two parallel *R*_ct_–CPE elements assigned to the anodic and cathodic interfacial charge-transfer processes, in addition to a terminal diffusion element (*W*) accounting for mass-transport limitations. In contrast, the anode spectrum (figure 11B) is adequately described by a simpler circuit composed of *R_s_*, one parallel *R_ct_***–**CPE element and a diffusion component, indicating that the isolated anode response is mainly controlled by anodic electron-transfer and diffusional transport. The use of a constant phase element (CPE) instead of an ideal capacitor is appropriate in both cases because the fabricated anode possesses a rough porous surface and becomes covered by a biofilm during operation, leading to nonideal interfacial capacitance and a distribution of relaxation times. The small depressed semicircle observed in the high-frequency region is therefore attributed to interfacial charge-transfer resistance, whereas the inclined low-frequency tail reflects diffusion of ions and electroactive species through the electrolyte, porous electrode structure and biofilm layer. Moreover, the need for an additional cathodic *R*_ct_–CPE branch in the whole-cell model confirms that the total cell impedance includes not only the anodic contribution but also the resistance associated with the air cathode and the overall reactor configuration.

#### 3.2.8. NaCl electrolyte

The effect of sodium chloride concentration on the performance of the MFC equipped with the 900°C graphitized palm-tree-based anode is presented in figure 12. The results in figure 12 show that the effect of NaCl concentration on MFC performance is not linear, and that only a moderate enhancement can be obtained by external NaCl addition when the 900°C anode is used. In general, the best performance was obtained at 6.25 g l^−1^ NaCl, whereas both 5.0 and 7.5 g l^−1^ NaCl produced considerably lower outputs. Thus, the performance order can be summarized as follows: 6.25 > 5.0 ≈ 7.5 g l^−1^ NaCl.

**Figure 12.**
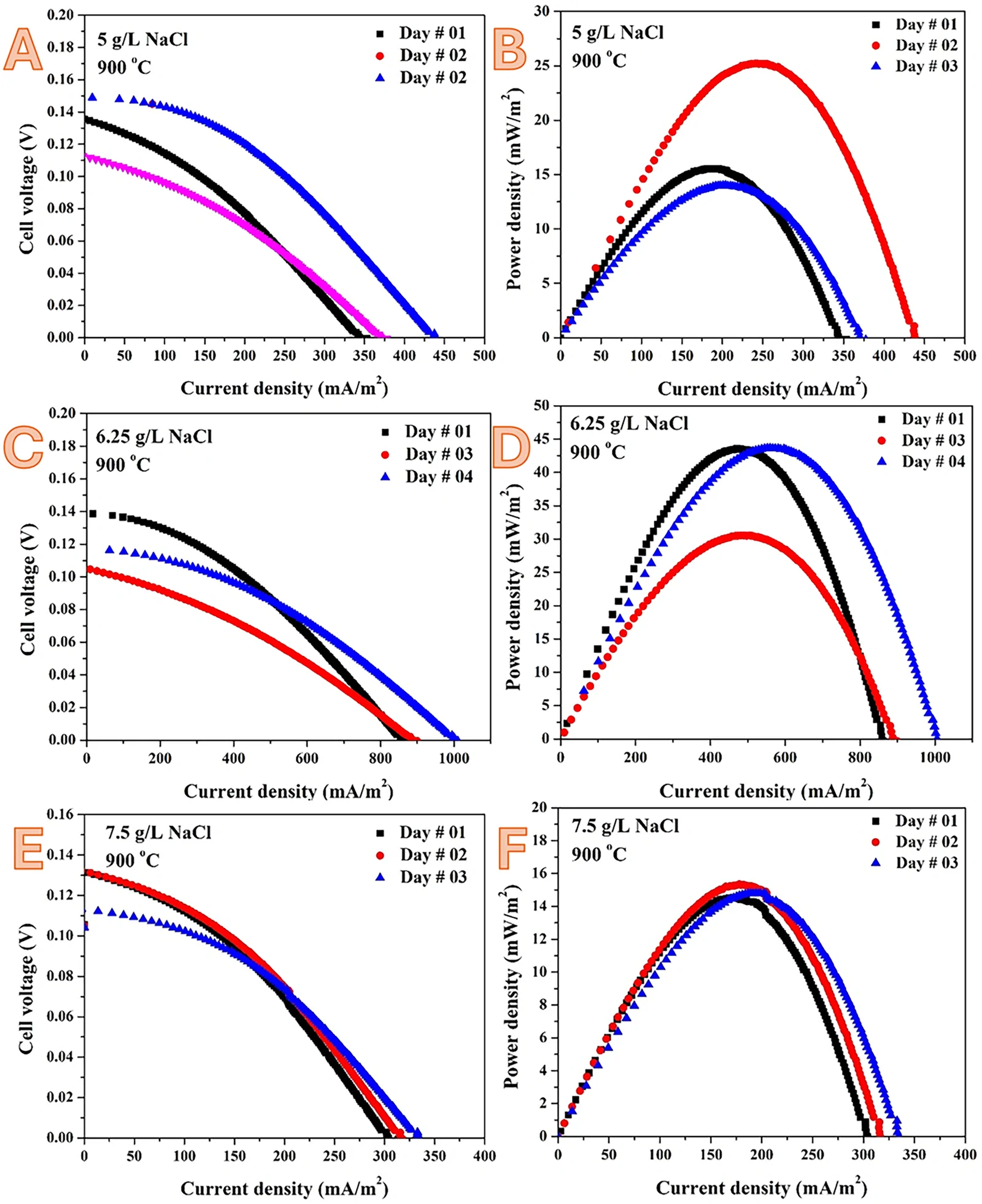
Effect of sodium chloride concentration on the performance of microbial fuel cells equipped with the graphitized palm-tree-branch-based anode prepared at 900°C. (A) Polarization curves at 5.0 g l^−1^ NaCl; (B) corresponding power-density curves at 5.0 g l^−1^ NaCl; (C) polarization curves at 6.25 g l^−1^ NaCl; (D) corresponding power-density curves at 6.25 g l^−1^ NaCl; (E) polarization curves at 7.5 g l^−1^ NaCl; and (F) corresponding power-density curves at 7.5 g l^−1^ NaCl.

At 5.0 g l^−1^ NaCl, the cell exhibited relatively low performance, with open-circuit voltage values in the range of about 0.11–0.15 V, maximum current density of about 0350–430 mA m^−2^ and maximum power density of approximately 14–25 mW m^−2^. A notable point here is that these values are very close to those obtained under the electrolyte-free condition for the same 900°C anode in figure 6, where the maximum power density also remained in the range of about 14–26 mW m^−2^. This observation is important because it indicates that adding 5.0 g l^−1^ NaCl did not produce a substantial further enhancement over the untreated wastewater system.

This apparent similarity between the electrolyte-free and 5.0 g l^−1^ NaCl systems can be reasonably explained by considering the actual composition of the electrolyte-free cell. Although no external salt was deliberately added in that experiment, the sewage wastewater itself already contained dissolved ionic species, and the graphitized anode released KCl, as confirmed by XRD analysis and the AgNO_3_ test. Therefore, the electrolyte-free system was not truly devoid of ionic support; rather, it already possessed a baseline ionic conductivity derived from both the wastewater and the intrinsic salt released from the anode. Under such conditions, the addition of 5.0 g l^−1^ NaCl may have provided only a limited extra increase in ionic strength, insufficient to cause a major rise in cell performance.

More importantly, the weak response of the 900°C system to NaCl addition suggests that the dominant limitation was not the bulk electrolyte conductivity alone. Earlier characterization results showed that the 900°C anode had the lowest electrical conductivity among the prepared graphitized palm-derived anodes and also exhibited the least favourable cyclic voltammetric behaviour. Accordingly, the performance of this electrode was likely controlled mainly by its relatively poor electron-transport properties and weak interfacial bioelectrochemical characteristics. In other words, the system was predominantly anode-limited rather than electrolyte-limited. As a result, improving the conductivity of the liquid phase by adding a low concentration of NaCl could not significantly enhance the overall power output.

A clear improvement was observed only when the NaCl concentration was increased to 6.25 g l^−1^. Under this condition, the maximum current density increased to about 840–1000 mA m^−2^, while the maximum power density rose to approximately 43–44 mW m^−2^. This represents nearly a 1.7-fold increase relative to the best value obtained at 5.0 g l^−1^ NaCl. Such enhancement indicates that, at this concentration, the increase in ionic strength became sufficient to reduce ohmic resistance more effectively and to improve ion migration through the cell. Thus, the 6.25 g l^−1^ NaCl condition appears to provide the most favourable electrolyte conductivity for this otherwise poorly conductive anode.

However, increasing the NaCl concentration further to 7.5 g l^−1^ did not produce additional benefit. Instead, the cell performance declined again, with maximum current density falling to about 310–340 mA m^−2^ and maximum power density being confined to only 14.5–15.2 mW m^−2^. These values are comparable to those of the electrolyte-free and 5.0 g l^−1^ NaCl cases, indicating that excessive NaCl had an adverse effect on the system. This decline can be attributed to the negative biological consequences of high salinity. While NaCl improves ionic conductivity, excessive salt concentration can impose osmotic stress on the indigenous microbial community, suppress metabolic activity and weaken biofilm-mediated extracellular electron transfer. Therefore, the positive electrolyte effect of NaCl at high concentration is likely offset by microbial inhibition.

The 900°C anode was selected for the NaCl-concentration study to evaluate whether increasing the ionic conductivity of the sewage medium could compensate for the comparatively poorer intrinsic conductivity and electrochemical characteristics of the lower-temperature graphitized electrode. This design allowed the contribution of bulk electrolyte conductivity to be distinguished from the intrinsic effect of anode structure and graphitization degree.

## 4. Conclusions

In this study, a simple and practical strategy was developed to fabricate self-supporting anodes from graphitized palm-tree-branch powder for membrane-less MFCs. The proposed method, based on powder blending with bitumen and starch followed by compression moulding and thermal treatment, successfully converted graphitized biomass into mechanically stable and electrochemically active bioanodes. The prepared anodes exhibited rough and porous surfaces favourable for microbial colonization, stable carbon-dominated electrochemical behaviour and higher electrical conductivity than the activated-carbon-based anode. Among the investigated materials, the anode prepared from palm tree branches graphitized at 1000°C showed the best overall performance, confirming that this temperature provided the most favourable balance between conductivity, surface accessibility and interfacial electrochemical activity. Under the optimum condition, using 5 g l^−1^ NaAc, the developed anode delivered a maximum power density of about 1300 mW m^−2^, which was nearly 25 times higher than that obtained with the activated-carbon-based anode. Even in electrolyte-free sewage wastewater, the same anode generated about 400 mW m^−2^, demonstrating that the system could sustain meaningful electricity generation without deliberate electrolyte addition. The results further indicated that intrinsically retained inorganic species, particularly KCl, may contribute to the ionic environment of the cell and partially support operation in the absence of external electrolyte. However, sodium acetate was markedly more effective than sodium chloride, highlighting that substrate availability and bioelectrochemical compatibility are more influential than ionic strength alone. EIS analysis confirmed relatively low ohmic and charge-transfer resistances under the optimum condition, while SEM analysis of the used anode suggested the formation of a dense biofilm-like layer on the electrode surface. Overall, from a practical perspective, the ability of the developed self-supporting anode to operate directly with sewage wastewater and indigenous microorganisms highlights its potential for simplified, decentralized wastewater treatment systems coupled with simultaneous bioelectricity recovery.

## Supplementary Material



## Data Availability

All data supporting the findings of this study are included within the text. Supplementary material is available online [45].
