# Supplementary material for: Self-supporting graphitized palm-branch anodes for enhanced electricity generation from sewage wastewater in membrane-less microbial fuel cells
Source: R Soc Open Sci. 2026 Oct 21;13(10):261016. doi: 10.1098/rsos.261016 (PMC13682878; doi:10.1098/rsos.261016)
Supplement: Supplementary file 1 [file rsos.261016.zip › rsos.261016/rsos261016_si_001.docx]

**Self-Supporting Graphitized Palm-Branch Anodes for Enhanced Electricity Generation from Sewage Wastewater in Membrane-Less Microbial Fuel Cells**

**Nasser A. M. Barakat^1,2,*^, Mahmoud M Bin Mahmoud^1,3^ and Ibrahim Ashour^1^**

^1^Chemical Engineering Department, Faculty of Engineering, Minia University, El-Minia 61516, Egypt

^2^Chemical Engineering Department, Faculty of Engineering, Atatürk University, 25240, Erzurum, Turkey

^3^Chemical Engineering Department, Faculty of Engineering, El-Mergib University, Al-Khums 40414, Libya

**Corresponding author**

**Nasser A. M. Barakat**

**Email:** [**nasbarakat@mu.edu.eg**](mailto:nasbarakat@mu.edu.eg)

**Tel: 0020 86 2321407**

**B**

**C**

**A**

**Fig. S01. Photographic images related to the fabrication of the molded anode: (A and B) specially designed stainless-steel mold used for shaping the anode, including the cylindrical powder-loading chamber, pressing rod, and bottom cover; (C) photograph of the final self-supporting anode prepared from graphitized palm tree branch powder after pressing and thermal treatment.**

**Table S01. Composition of the prepared anodes fabricated from graphitized palm tree branch powder, bitumen, and starch at different graphitization temperatures (900, 1000, and 1100 °C), together with the optimized formulation used to obtain suitable mechanical properties.**

|  | **Graphitization Temperature** | **Functional Material (g)** | **Bitumen**  **(g)** | **Starch**  **(g)** |
| --- | --- | --- | --- | --- |
| **Palm tree branch** | **900** | **12** | **10** | **3** |
|  | **1000** | **15** | **20** | **15** |
|  | **1100** | **10** | **15** | **5** |
| **Activated carbon** |  | **15** | **15** | **5** |

**A**

**C**

**B**

**Fig. S02. Photographic images for the separated cell components; (A) and the assembled cell from two sides; (B and C)**

**Fig. S03 Videoclip for formation of white precipitate (AgCl) upon addition of drops from AgNO_3_ solution.**
